# New insights into the diversity of cryptobenthic *Cirripectes* blennies in the Mascarene Archipelago sampled using Autonomous Reef Monitoring Structures (ARMS)

**DOI:** 10.1002/ece3.9850

**Published:** 2023-03-16

**Authors:** Marion Couëdel, Agnes Dettai, Mireille M. M. Guillaume, Fleur Bruggemann, Sophie Bureau, Baptiste Frattini, Amélie Verde Ferreira, Jean‐Lindsay Azie, J. Henrich Bruggemann

**Affiliations:** ^1^ Université de La Réunion, UMR 9220 ENTROPIE (Université de La Réunion, IRD, IFREMER, Université de Nouvelle‐Calédonie, CNRS) La Réunion Saint‐Denis France; ^2^ Muséum national d'Histoire naturelle (MNHN), UMR 7205 ISYEB (MNHN, CNRS, Sorbonne Université, EPHE, Université des Antilles) Paris France; ^3^ Muséum national d'Histoire naturelle (MNHN) UMR 8067 BOrEA (MNHN, CNRS 2030, Sorbonne Université, IRD 207, Uni Caen‐Normandie, Université des Antilles) Paris France; ^4^ LabEx CORAIL Université de Perpignan Perpignan France; ^5^ Rodrigues Regional Assembly Port Mathurin Rodrigues Mauritius

**Keywords:** barcoding, coral reefs, cryptic teleosts, mitogenome, molecular species delineation, South‐West Indian Ocean

## Abstract

Autonomous Reef Monitoring Structures (ARMS) are artificial mini‐reefs designed for standardized sampling of sessile and small motile cryptobenthic organisms. ARMS are also effective for collecting small cryptobenthic fishes, such as the combtooth blennies of the genus *Cirripectes*. Recent studies discovered several *Cirripectes* species endemic to islands or archipelagos, in spite of the generally broad distributions of tropical and subtropical blennies. Thus, to evaluate the diversity and distribution of *Cirripectes* species in the Mascarene Archipelago, a little‐studied region but an important biodiversity hotspot, complete mitochondrial genomes, and nuclear rhodopsin genes were sequenced for 39 specimens collected with ARMS deployed on outer reef slopes at Reunion and Rodrigues islands. Mitochondrial COI sequences were analyzed to integrate these specimens within the largest dataset of publicly available sequences. Three species were found in the Mascarene Archipelago, *Cirripectes castaneus, Cirripectes randalli*, and *Cirripectes stigmaticus*. *C. castaneus* and *C. stigmaticus* both have an Indo‐Pacific distribution with several haplotypes shared among distant localities. In agreement with the literature, *C. randalli* shows a small‐range endemism restricted to the Mascarenes. We confirmed the presence of *C. castaneus, C. randalli*, and *C. stigmaticus* in Rodrigues, and the presence of *C. stigmaticus* in Reunion. This study contributes to filling the gaps in taxonomic and molecular knowledge of the reef cryptobiome in the South‐West Indian Ocean, and provides the first complete mitogenomes for the genus, a crucial step for future molecular‐based inventories (e.g., eDNA).

## Graphical abstract

This study uses Autonomous Reef Monitoring Structures (ARMS) and multiplexed NGS sequencing of long amplicons to investigate phylogeographic relationships within combtooth blennies *Cirripectes*. Our results confirm the presence of *C. castaneus*, *C. randalli*, and *C. stigmaticus* in Reunion and Rodrigues and contribute to filling the gaps in taxonomic and molecular knowledge of reef cryptobiome for the South‐West Indian Ocean. Our molecular approach provides the first complete mitogenomes for three species and the *Cirripectes* genus.

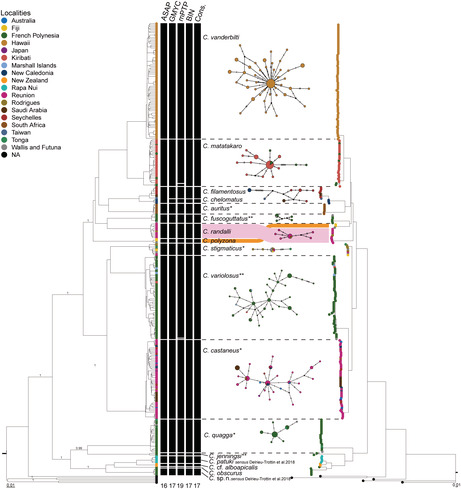

## 1 INTRODUCTION

Understanding coral reef ecosystem functioning requires knowledge of the distribution and abundance of reef‐associated fishes. Due to their accessibility to visual observation, the larger reef fishes have been intensively studied for decades (Knowlton et al., 2010). Although their diversity and taxonomy appeared relatively well resolved (Allen, 2015; Fisher et al., 2015; Mora et al., 2008), molecular studies revealed extensive hidden diversity (Hubert et al., 2017; Steinke et al., 2009). The smaller taxa present additional challenges, as they are inherently more difficult to find and identify, and are therefore often omitted from visual surveys and collections (Bellwood et al., 2019; Brandl et al., 2018; Pearman et al., 2018). Although they are often overlooked, their distinctive demographic dynamics may make them a cornerstone of ecosystem functioning in modern coral reefs (Brandl et al., 2019).

Cryptobenthic reef fishes are small, bottom‐dwelling, morphologically, or behaviorally cryptic species. They comprise families such as combtooth blennies (Blenniidae), gobies (Gobiidae), triplefins (Tripterygiidae), and cardinalfishes (Apogonidae). Despite being the ocean's smallest vertebrates, they contribute disproportionately to coral reef food webs through their high abundance, rapid somatic growth, and high predation mortality, producing almost 60% of reef fish biomass consumed within the ecosystem (Brandl et al., 2019). Furthermore, cryptic fishes represent approximately 10% of vertebrate diversity on coral reefs and can exhibit high levels of endemism (Bellwood et al., 2019; Brandl et al., 2018).

The genus *Cirripectes* Swainson, 1839 (Family Blenniidae, Order Blenniiformes) comprises 24 recognized species of combtooth blennies, broadly distributed in the Indo‐Pacific from East Africa to Rapa Nui in the eastern Pacific (Hastings & Springer, 2009; Hoban & Williams, 2020; Williams, 1988). Currently, 14 species are recorded with COI DNA sequences in the BOLD database (Ratnasingham & Hebert, 2007), plus three genetically divergent groups that possibly represent not yet described new species. Most *Cirripectes* species are smaller than 100 mm. They are herbivorous and/or detrivorous cryptobenthic teleosts that primarily inhabit rocky or coral substrates in shallow (<5 m depth) high‐surge fore reef habitats (Williams, 1988). However, individuals of *Cirripectes matatakaro* and *Cirripectes castaneus* may be encountered deeper (more than 20 m and over 32 m depth, respectively; Williams, 1988, Hoban & Williams, 2020). Species show considerable variation in geographic range sizes, from small area endemism (e.g., *Cirripectes heemstraorum* endemic to the East coast of South Africa at Cape Vidal; Williams, 2010) to Indo‐Pacific‐wide distributions (e.g., *C. castaneus*; Williams, 1988). Blennies' eggs are demersal and attached to the substratum with a filamentous, adhesive pad or pedestal (Breder & Rosen, 1966; Watson, 2009). Larvae are planktonic and abundant in shallow, coastal waters (Watson, 2009). While *Cirripectes* specimens are common in museum collections, incomplete knowledge of sexual dimorphism and geographic color variations, combined with a lack of adequate species identification keys, have resulted in numerous misidentifications and undetected cryptic species (Williams, 2010). In many cases, color morphs were considered distinct species, even though sexual polychromatism has been described for several *Cirripectes* species (Williams, 1988).

The latest fish checklist of Reunion reported six species of *Cirripectes* (Wickel et al., 2020): *C. castaneus*, *Cirripectes filamentosus*, *Cirripectes polyzona*, *Cirripectes quagga, Cirripectes randalli*, and *Cirripectes stigmaticus* (Table 1). For Rodrigues, four *Cirripectes* species have been listed: *C. castaneus*, *C. filamentosus*, *Cirripectes gilberti* and *C. stigmaticus* (Heemstra et al., 2004). *Cirripectes auritus* was not recorded in Reunion and Rodrigues but was described as present in Mauritius (Debelius, 1993; Fricke, 1999). Most of these *Cirripectes* species are considered to be widely distributed. *Cirripectes filamentosus*, *Cirripectes polyzona*, *Cirripectes quagga*, and *C. stigmaticus* occur throughout the Indo‐Pacific, while the distribution of *C. auritus* and *C. castaneus* ranges from East Africa to the western Pacific. *C. gilberti* has a more restricted distribution and occurs throughout the Indian Ocean (Williams, 1988). However, recent studies discovered several *Cirripectes* species endemic to islands or archipelagos (Delrieu‐Trottin et al., 2018; Hoban & Williams, 2020). For the Mascarenes, only *C. randalli* is known to have a distribution limited to the archipelago (Williams, 1988). Ecological and geographical distributions of these species are summarized in Table 1.

**TABLE 1:** *Cirripectes* species reported from the Mascarene Archipelago with their ecological and geographical distributions.

Species	Habitat	Depth	Polymorphisms	Distribution
*C. auritus* (Carlson, 1981)	Coral reefs	<10 m; max 20 m	S + G	Indo‐West Pacific
*C. castaneus* (Valenciennes, 1836)	Rocky and coralline substrates; wave‐swept algal ridges	<10 m; max 30 m	S + G*	Indo‐West Pacific
*C. filamentosus* (Alleyne & Macleay, 1877)	Coral and rocky reefs; tolerate a wider range of environmental conditions than other *Cirripectes* spp	<7 m; 20 m	S (+G)	Indo‐West Pacific
*C. gilberti* (Williams, 1988)	Rocky and coralline substrates	<8 m	S	Indian Ocean
*C. polyzona* (Bleeker, 1868)	Algal ridges and crests between surge channels of exposed seaward reefs	Usually <3 m; <20 m	S	Indo‐Pacific
*C. quagga* (Fowler & Ball, 1924)	Algal ridges and crests between surge channels of exposed seaward reefs	<10 m; max 19 m	S + G*	Indo‐Pacific
*C. randalli* (Williams, 1988)	Coral patches in surge channels of rocky reefs with light surf	<8 m	S	Mascarenes
*C. stigmaticus* (Strasburg & Schultz, 1953)	Upper edge of seaward reef slopes. Adults inhabit coastal reef flats with rich corals and algae. Among *Acropora* and *Pocillopora* corals of wave‐swept algal ridges	<20 m	S + G	Indo‐Pacific

*Note*: The types of polymorphism were indicated as ‘S’ for sexual and ‘G’ for geographical. Species with more than one sympatric sexual color pattern are indicated by *. Information was synthetized from Williams (1988), Letourneur et al. (2004), and Allen et al. (2013).

Cryptobenthic fishes are often under‐sampled due to their hidden habits. Therefore approaches focused on sampling small and cryptobenthic fauna, such as artificial mini‐reefs ARMS, represent alternative sampling techniques. ARMS, for Autonomous Reef Monitoring Structures, are stacks of nine PVC plates spaced at a 12 mm distance, designed to mimic the complexity of coral reef habitats. Each ARMS represents slightly over 4.5 L of habitat volume. Affixed to the seabed, they are left to be colonized by a diversity of marine species, then collected and dismantled to study the associated biota (see Zimmerman & Martin, 2004 for more details).

To evaluate the diversity and distribution of *Cirripectes* species in the Mascarene Archipelago, a little‐studied region with high endemism and source of type material for this genus, we reconstructed the phylogeographic relationships within *Cirripectes* collected using ARMS. We conducted a multi‐marker approach by sequencing mitochondrial and nuclear genes and integrated newly collected specimens within the largest dataset of publicly available sequences. We sequenced complete mitochondrial genomes as part of the current effort to complete the inventory of teleosts in French territories led by the *Muséum national d'Histoire naturelle* (MNHN). Having available complete mitogenomes enables the construction of more robust phylogenies, but the current paucity of mitogenomes in public databases makes such analyses premature. Finally, we explored the nucleotide divergences between *Cirripectes* species found in the Mascarene Islands to assess the performance of current mini‐barcodes used to detect teleosts species in eDNA studies.

## 2 MATERIALS AND METHODS

### 2.1 Specimen collection

Specimens were collected from 54 ARMS deployed between September 2014 and August 2021 at two islands of the Mascarene Archipelago (South‐West Indian Ocean): along the western and south‐western coasts of Reunion Island (10 sites) and along the North coast of Rodrigues (3 sites). At each site, three replicate ARMS units were deployed on spurs of outer coral reef slopes at 10–12 m depth, with immersion times varying from 6 months to 4 years. The ARMS deployment recovered 144 fishes, of which 39 specimens were *Cirripectes* (Appendices S1 and S2). These samples comprised at least four teleost families, including 85 Gobiidae (mainly *Eviota* and *Enneapterygius*), 40 Blenniidae (39 *Cirripectes*, 1 *Aspidontus*), 2 Pomacentridae (*Pycnochromis nigrurus*), 2 Muraenidae (including one *Gymnothorax*), and 15 specimens of undetermined affinity. Most individuals were photographed alive, identified to the lowest taxon level possible based on morphology, individually preserved in 90% ethanol (EtOH) and stored at 4°C. For *Cirripectes* specimens, the total length was measured with a ruler (Appendix S2).

### 2.2 DNA extraction and sequencing

Muscle tissue was used for total genomic DNA extraction using DNeasy Blood & Tissue Kit (Qiagen), following the manufacturer's instructions. PCR amplification and sequencing were performed for the entire mitochondrial genome and the partial retro‐rhodopsin nuclear gene (Rh193 and Rh1039r; Chen et al., 2003). The mitogenome was amplified in three overlapping fragments. The first fragment from the end of the 16S to the end of COI was amplified with 16SAR (Kocher et al., 1989) and MtH7061 (Hinsinger et al., 2015). The second fragment from the beginning of COI to the end of ND4 was amplified with F5231cha (Hinsinger et al., 2015) and MtH11944 (Hinsinger et al., 2015). Additionally, we developed three new primers, R11944cha 5′‐CATAGCTNCTACTTGGATTTGCACCA‐3′ and two specific primers designed for the genus *Cirripectes*: F5231Cirri 5′‐TAGRCAGGCAGGCCTCGATCCTRCA‐3′ and R11944Cirri 5′‐CATAGTTTCTGCTTGGAGTTGCACCA‐3′ to improve amplification success. The third fragment from ND5 to the end of 16S was amplified with MtL11910 (Hinsinger et al., 2015) and 16SBR (Kocher et al., 1989). The amplicons were pooled with other PCR amplicons following Hinsinger et al. (2015) for cost efficiency. Library preparation followed Meyer and Kircher (2010) and Illumina MiSeq sequencing (PE250) was performed at the *Service de Systématique Moléculaire* of the MNHN at Concarneau and at the *Institut du Cerveau et de la Moëlle Epinière* (Pitié‐Salpêtrière Hospital, Paris). Two rhodopsin samples were sequenced in both directions through Sanger sequencing by Eurofins Genomic, France.

### 2.3 Sequences processing and public sequences retrieval

**FIGURE 1:**
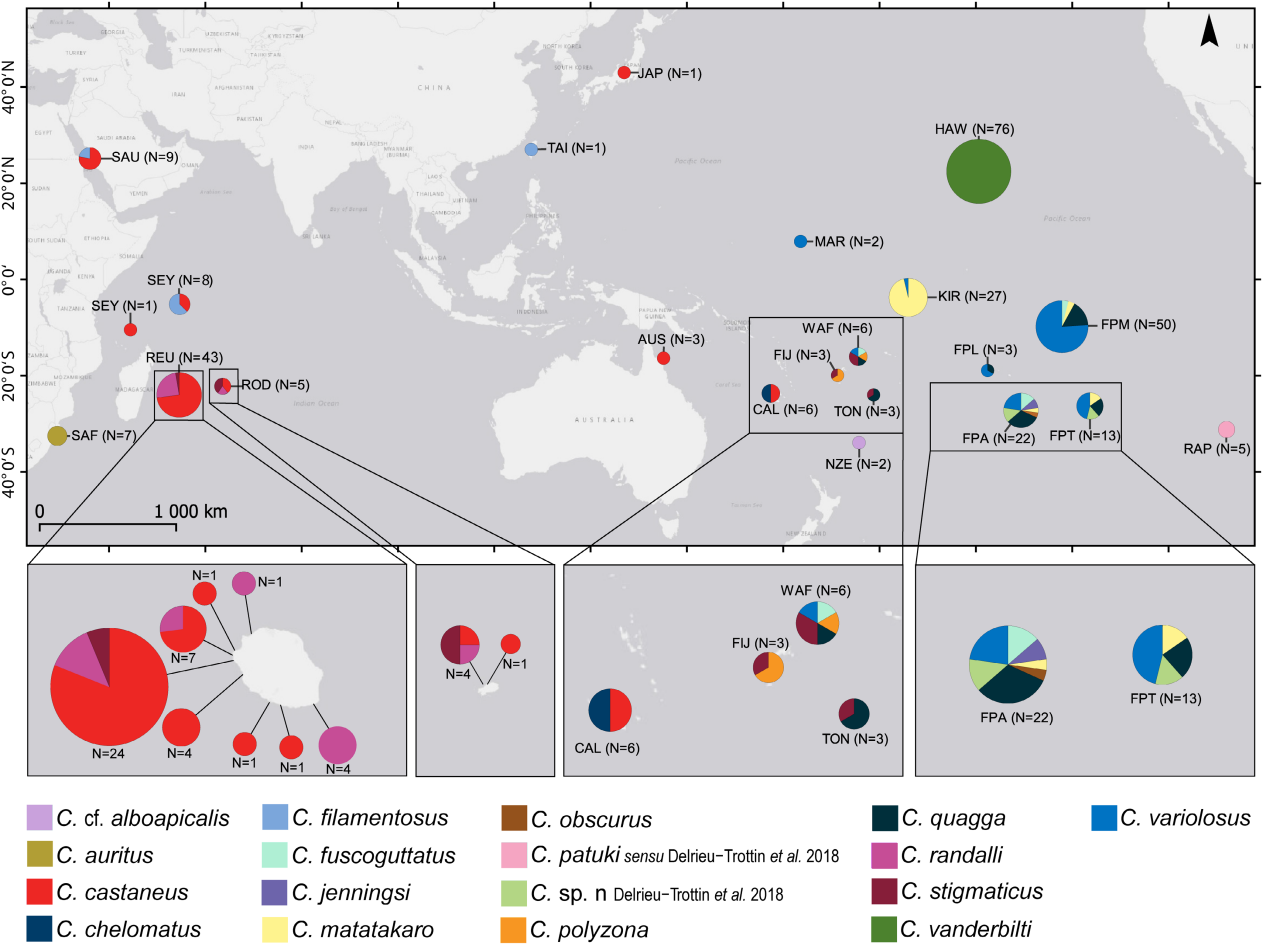
*Cirripectes* species distribution inferred from the COI dataset based on molecular delimitation analyses. AUS, Australia; CAL, New Caledonia; FIJ, Fiji; FPA, French Polynesia, Austral Islands; FPL, French Polynesia, Leeward Islands; FPM, French Polynesia, Marquesas and Society Islands; FPT, French Polynesia, Tuamotu‐Gambier; HAW, Hawaii; JAP, Japan; KIR, Kiribati; MAR, Marshall Islands; NZE, New Zealand; RAP, Rapa Nui; REU, Reunion; ROD, Rodrigues; SAF, South Africa; SAU, Saudi Arabia; SEY, Seychelles; TAI, Taiwan; TON, Tonga; WAF, Wallis and Futuna. The size of pie charts is proportional to the number of samples.

Reads were processed with Geneious Prime 2019.2.3. Paired‐end reads were merged, the primers were used as barcodes to recover the fragment ends and the merged reads were de novo assembled. The resulting contigs were checked in BOLD and Genbank databases before being used as references for elongation through repeated mapping of reads with a maximum of 1% mismatch and three bases gap allowed. Mapping was repeated until no further reads could be mapped. Complete linear mitochondrial consensuses were transformed into circular sequences and overlapping sections were manually inspected and adjusted. Last, reads were mapped back against the obtained circular sequences to check coverage and final assembly. The mitogenome sequences were annotated using online MitoFish (Iwasaki et al., 2013). Sanger sequences were assembled and checked in Geneious Prime 2019.2.3 (2019). To increase taxonomic and spatiotemporal coverage, our datasets were extended with publicly available sequences from BOLD and/or GenBank (Figure 1; ESM 1). In some instances, sequences were renamed according to the conclusions of the papers in which they were published, even if the names were not corrected in the database: sequences within BOLD BIN:AAU0601 were renamed from *C. castaneus* to *C. randalli* (Hoban & Williams, 2020), and MH932003 to MH932007 were renamed from *C. alboapicalis* to *C. patuki* sensu Delrieu‐Trottin et al., 2018 (Delrieu‐Trottin et al., 2018). Two sequences, GBMNB4802‐20 and KX223895.1, respectively from BOLD and Genbank, probably belong to a different genus and were removed from the analyses.

### 2.4 Molecular and phylogenetic analyses

All analyses were performed on three datasets: (i) mitochondrial cytochrome oxidase I (further called COI dataset; *N* = 296; ESM 1), (ii) nuclear rhodopsin (further called Rho dataset; ESM 2) for which 53 sequences were used corresponding to 17 homozygotes and 2 × 18 heterozygotes (NGS reads were checked for gene versions); for haplotype analyses, 70 sequences were used corresponding to 35 specimens with two alleles, and (iii) a dataset consisting of the concatenated two rRNA (12S and 16S) and 13 coding DNA sequence (CDS: ND1, ND2, COI, COII, ATPase 8, ATPase 6, COIII, ND3, ND4L, ND4, ND5, ND6, and Cyt b) from the complete mitogenome (henceforth called mt dataset; *N* = 24). Based on the Blenniidae phylogeny of Lin and Hastings (2013), we selected sequences from multiple outgroup species (COI: *N* = 5; Rho: *N* = 4; mt: *N* = 4) from the recognized tribes *Ophioblennius macclurei*, *Omobranchus elegans*, *Omobranchus obliquus*, *Petroscirtes breviceps*, *Salarias fasciatus*, and *Ecsenius bicolor* (Appendix S3). Sequences of each marker were aligned separately via Muscle 3.8.425 (Edgar, 2004), implemented in Geneious Prime 2019.2.3 using default parameters, and manually trimmed to maximize the shared length among the sequences (COI = 506 bp, Rho = 737 bp). For the mt dataset, complete mitogenomes were aligned and translated into protein to check the codon position for the coding genes. The two tRNA and 13 CDS were kept according to coding position and overlapping CDS portions were trimmed at the end to prevent a shift in the translation frame.

ModelFinder 1.6.8 (Kalyaanamoorthy et al., 2017) in PhyloSuite (Zhang et al., 2020) was used to select the evolutionary model with the best Akaike Information Criterion (AIC; Akaike, 1974) for each dataset. Maximum‐likelihood (ML) and Bayesian inference (BI) analyses were conducted. ML tree searching was conducted in W‐IQ‐TREE (Trifinopoulos et al., 2016) for 1000 ultrafast bootstraps (Hoang et al., 2018). Multiple independent Bayesian inference searches used strict or uncorrelated log‐normal relaxed molecular clock models with a Yule or birth rate ratio tree prior in BEAST2 2.1.2 (Bouckaert et al., 2014). Codon partitions gene and chains were run for 10 million generations, using Tracer (Rambaut et al., 2018) to confirm stationarity and mixing. Maximum Clade Credibility (MCC) tree was obtained through TreeAnnotator 1.10.4 and the generations before reaching stability were discarded as burn‐in. For the mt dataset, TreeAnnotator did not allow to recover an MCC tree, which is consistent with the lack of convergence observed in Tracer. Therefore Bayesian inference was computed with MrBayes 3.2.6 (Ronquist & Huelsenbeck, 2003) in PhyloSuite (Zhang et al., 2020). The reconstruction was visualized in R 4.1 (R Core Team, 2021).

Three molecular delineation approaches were run separately on the COI, rhodopsin, and mitochondrial concatenated datasets: (i) Assemble Species by Automatic Partitioning (ASAP; Puillandre et al., 2021), (ii) the multi‐rate Poisson Tree Processes (mPTP) method (Zhang et al., 2013), and (iii) a single threshold General Mixed Yule‐Coalescent (GMYC) approach (Fujisawa & Barraclough, 2013) implemented with the R 4.1 package “splits” (Ezart et al., 2009; R Core Team, 2021). Additionally, Refined Single Linkage (RESL) analysis was performed on the COI dataset. RESL is used for the barcode identification numbers (BINs) system implemented in BOLD. The relationships among haplotypes from distinct localities were inferred from haplotype networks built using median‐joining (Bandelt et al., 1999) methods implemented in popART 1.7 (Leigh & Bryant, 2015). Nucleotide diversity, Tajima's D, and an AMOVA were conducted in Arlequin 3.5 (Excoffier & Lischer, 2010), following the different clades observed in phylogenetic trees, to assess the level of genetic differentiation within and between the groupings obtained.

### 2.5 Sliding window analyses

Sliding window analyses were performed to explore nucleotide divergence between *Cirripectes* species found in the Mascarene Islands and to determine the performance of current mini‐barcodes used to detect teleosts species in eDNA (MiFish [163–185 bp; Miya et al., 2015]; Teleo [65 bp; Valentini et al., 2016]). These analyses were performed using the R package “SPIDER” 1.1.2 (Brown et al., 2012) with a window size of 160 and 65 bp for the MiFish and the Teleo markers, respectively, and with a step size of 1 bp. Divergence analyses were also performed on complete COI and partial COI fragments from Geller et al., 2013 (jgLCO1490‐jdHCO2198) which are mostly used for metabarcoding of broad taxonomic groups.

## 3 RESULTS

### 3.1 Sequence analysis

The specimens were deposited in the MNHN fish collections under collection numbers MNHN‐IC‐2023‐0216 to MNHN‐IC‐2023‐0254. The sequences produced in this study were deposited in the BOLD dataset “Cryptic fishes from IO ARMS” (IOACT) and in Genbank under accession numbers presented in Appendix S2; the specimens were deposited in the collections of the MNHN. We generated 34 complete COI sequences representing 22 haplotypes. Including public sequences from BOLD and GenBank databases, the COI dataset was composed of 296 sequences of 506 bp from 15 species names and two undescribed groups, including 15 public BOLD BINs and 18 localities (Figure 1). The COI dataset comprised 135 haplotypes (ambiguities or missing data were not taken into account; if considered *N* = 159) with 170 parsimony informative sites. Out of 22 haplotypes, 15 were new while the remaining seven were identical to previously published sequences. A total of 176 bp of the 506 bp of the COI region were variable (34.78%; Appendix S4). The third codon position of COI sequences provided significantly more parsimony informative sites (89.88% were informative) than the first (11.31%) and the second (0%) codon positions.

The 27 rhodopsin sequences obtained by NGS were checked for heterozygotes. This yielded 14 distinct sequences. The complete dataset including public sequences comprised 70 sequences for 35 individuals (corresponding to six species names and five localities) and contained a total of 25 haplotypes, 11 from public databases and 14 from this study (13 new). The rhodopsin dataset had 30 variable sites (4.07%) and 19 parsimony informative characters (2.58%) over 737 bp (*π* = 0.00479).

The mitogenome dataset comprised complete mitochondrial genomes of 18 *C. castaneus*, 2 *C. randalli*, and 4 *C. stigmaticus*. For 10 *C. castaneus*, mitogenomes were not recovered completely. Out of the five partitions, the third codon position of the CDS partition provided significantly more parsimony informative sites (49.39%) than the first and second positions, or the two tRNA partitions (15.69% and 26.84%). The 12S sequence had the lowest proportion of both variable characters (29.21%) and parsimony informative characters (15.69%).

### 3.2 Phylogenic reconstruction of *Cirripectes*

For the COI dataset, the nucleotide substitution model with codon partition had less good fit than the single model; hence, the GTR + F + I + G4 model was selected for the analysis (Appendix S4). For the mitochondrial concatenated dataset sequences, the best‐fit partitioning scheme was GTR + I + G4. For rhodopsin, different partition models were used. While TVM + F + G was the best‐fit partitioning scheme for the first codon position, this scheme was unfortunately not implemented in BEAST. However, it was equivalent to the GTR + F + I + G4 model with fixed AG rate parameters (1.0; Bagley, 2018). For the second and third codon positions, F81 + F + G4 and GTR + F + G4 were respectively selected.

**FIGURE 2:**
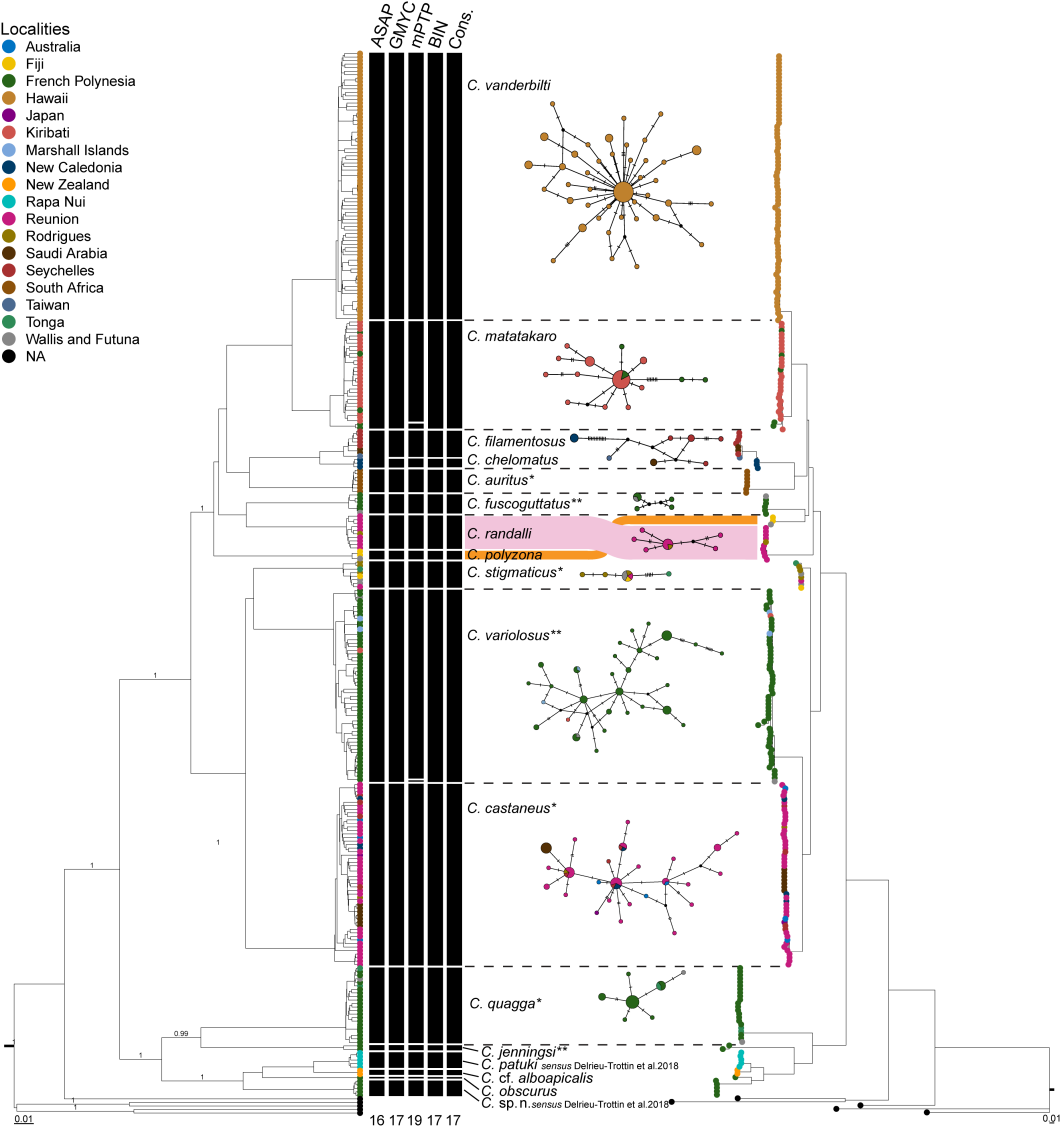
Ultrametric BEAST Bayesian Inference (left) and Maximum‐Likelihood (right) trees of COI mitochondrial dataset, with a graphical representation of the results of the ASAP, GMYC, and mPTP molecular species delimitation analyses, BIN clustering from BOLD (RESL), and the resulting consensus clusters (Cons.). Between trees, median‐joining networks for the consensus clusters. Groups with ≤3 haplotypes are not shown, except for *C. chelomatus* which is very close to *C. filamentosus*. Each circle represents a haplotype with size proportional to total frequency. Black crossbars on branches represent single nucleotide changes, black nodes indicate non‐sampled probable haplotypes, while colors denote collection location as indicated in the embedded key. *Indicates species described as having Indo‐Pacific ranges while **denotes species with Pacific ranges (from Williams, 1988).

Maximum‐likelihood and BI‐based phylogenetic reconstructions for COI resulted in tree topologies with marked similarities (Figure 2). For the 17 species in the dataset, the COI trees recovered well‐supported clades within the genus. However, the branching order of these clades presented differences among methods. Clades 6 and 7 were sister species in the BI analysis, while clade 6 was closer to clade 8 within the ML approach. These incongruent results were probably related to the short branch lengths at the base of clades 1–8. *Cirripectes* appear to be a monophyletic genus relative to outgroups (Figure 2). *Cirripectes* collected in the Mascarenes were present in three clusters within two separate larger clades of the trees (Figure 2). For the mitochondrial dataset, both ML and BI trees strongly supported the grouping of our specimens in three clades (Appendix S5). For the rhodopsin dataset, both BI and ML trees had poorly supported clades (not shown) probably because of the low variability of sequences (1.63–15.92% of parsimony informative sites; Appendix S4).

### 3.3 Molecular species delimitation

Based on the COI dataset, the ASAP molecular species delimitation approach divided the sequences into 16 groups, while the GMYC and BIN subdivided the COI dataset into 17 groups, and mPTP subdivided into 19 groups (Figure 2; ESM 1). For the sequences from specimens identified as *C. filamentosus* and *Cirripectes chelomatus*, GMYC, BIN, and mPTP methods divided sequences according to geographic origin (the western Indian and western Pacific oceans, respectively) and distinct morphological characters (Williams, 1988). The mPTP method was the only one that subdivided specimens identified as *Cirripectes matakaro* into two groups, as was the case for those identified as *Cirripectus variolosus*. These two subdivisions were not supported by the other methods and they will not be considered further here. Therefore, a final delimitation scheme was established based on a majority‐rule consensus among the different delimitation analyses which included the 16 clades from the ASAP approach and the *C. chelomatus* clade delimited by the three other methods. Most of these can be unequivocally associated with *Cirripectes* species names from public databases and literature, except for clade 17 (*Cirripectes* sp. n. Delrieu‐Trottin et al., 2018; Figure 2; ESM 1). According to Hoban and Williams (2020), clade 11 corresponded to *C. castaneus* and clade 9 to *C. stigmaticus* (ESM 1). However, sequences from the group named *C. castaneus* contained samples identified as *C. castaneus* and others as *C. stigmaticus* in public databases.

### 3.4 Sequence variability and haplotype relationship

Nucleotide diversity for the COI marker was low and ranged from 0.000 to 0.014 (Table 2). *Cirripectes* showed an average difference of 2.07 bp (0.41%; 0–1.38%) within species and 61.43 bp (11.98%; 2.60–18.77%) among species (Appendix S6). *C. castaneus* and *C. stigmaticus* had a larger difference average of 5.38% (27.2 bp).

**TABLE 2:** Genetic diversity measures of *Cirripectes* species.

Species	# Sequences		# Haplotypes		# Polymorphic sites		# Nucleotide diversity		Differentiation for COI (%)		Tajima's D	
	COI	Rho	COI	Rho	COI	Rho	COI	Rho	Intra‐specific	Inter‐specific	COI	Rho
*C*. cf. alboapicalis*	2	–	1	–	0	–	0	–	–	4.09	0	–
C. auritus*	7	–	3	–	2	–	0.00111	–	0.11	8.60	−1.23716	–
*C. castaneus*	51	52	30	15	25	15	0.005411	0.001325	0.55	4.91	−1.80603*	2.15061*
C. chelomatus*	3	6	2	3	1	2	0.00129	0.001447	0.13	2.60	0.00000	1.03194
*C. filamentosus*	8	2	7	2	11	1	0.00758	0.001357	0.75	3.04	−0.95806	0
*C. fuscoguttatus*	6	–	4	–	5	–	0.00414	–	0.42	8.04	−0.14427	–
C. jenningsi*	2	–	2	–	7	–	0.01359	–	1.38	8.85	0.00000	–
*C. matatakaro*	31	–	14	–	21	–	0.00431	–	0.44	3.92	−2.01227*	–
C. obscurus*	1	–	1	–	0	–	–	–	–	7.09	0.00000	–
*C. patuki* sensu Delrieu‐Trottin et al., 2018*	5	–	5	–	7	–	0.005929	–	0.59	4.39	−0.74682	–
*C. polyzona*	3	–	2	–	2	–	0.00259	–	0.26	8.10	0.00000	–
*C. quagga*	22	2	7	2	7	1	0.00247	0.001357	0.25	12.35	−1.07910	0
*C. randalli*	10	2	6	1	8	0	0.00341	0	0.35	8.71	−1.63600*	0
*C. stigmaticus*	8	6	4	3	7	4	0.00374	0.001809	0.38	5.38	−1.35929	−1.29503
C. vanderbilti*	76	–	40	–	42	–	0.00410	–	0.41	4.34	−2.45250*	–
*C. variolosus*	55	–	29	–	35	–	0.00827	–	0.83	6.46	−1.49064*	–
*C*. sp. n. Delrieu‐Trottin et al., 2018*	5	–	2	–	1	–	0.000791	–	0.08	7.75	−0.81650	–
Global	296	70	159	26		30						

*Note*: Species with * have been sampled from one locality only.

The AMOVA supported the hypothesis of the above species grouping and high levels of genetic structure, with Fst = 0.94 (*p* < .001) for COI. Moreover, a high percentage variation (94.59%) was observed for COI among species while a low percentage variation (5.41%) occurred within species (Appendix S7). Population pairwise tests also revealed that COI haplotypes were significantly genetically different in 113 of 136 comparisons (*p* < .05; Appendices S8 and S9). Most non‐significant values were obtained for comparison with *Cirripectes obscurus* due to the insufficient number of sequences (*N* = 1; Table 2). For rhodopsin, the AMOVA results also supported the above species grouping (for species present in the dataset: Fst = 0.86; *p* < .001; Appendix S7).

The haplotype networks based on COI sequences were computed for each of the 17 species from the molecular delineation analyses (Figure 2). *Cirripectes* species presented several different haplotype network structures. *Cirripectes vanderbilti* had a star‐shaped network, with one abundant haplotype surrounded by less common haplotypes, indicative of a possible recent population expansion or high demographic turnover within Hawaii (Grant & Bowen, 1998; Hoban & Williams, 2020). In contrast, *C. castaneus* had a complex star network with several abundant haplotypes surrounded by less common ones. Only the haplotype networks from species collected in this study are detailed here. The *C. castaneus* group comprised a higher number of specimens (Ns = 52) and haplotypes (Nh = 30), compared to the *C. randalli* (Ns = 10; Nh = 6) and *C. stigmaticus* (Ns = 8; Nh = 4; Table 2) groups. The *C. castaneus* network showed a unique haplotype for specimens collected in the Red Sea. *C. castaneus* haplotypes had wide geographic ranges with one haplotype shared among western Indian Ocean localities (Reunion, Rodrigues, Seychelles) and three shared among Indo‐South Pacific localities (two by Reunion, Seychelles, New Caledonia and one by Reunion and Australia; Figure 2). The *C. randalli* network displayed one haplotype common to both Mascarene localities (only one specimen for Rodrigues) and one *C. stigmaticus* haplotype was shared among Indo‐South Pacific localities (Reunion, Rodrigues, Fiji, Wallis & Futuna, and Tonga). Compared to other species included in the dataset, only *C. castaneus* and *C. stigmaticus* contained specimens from Indo‐Pacific localities. The haplotype network computed on the rhodopsin dataset generated similar but less detailed information in terms of haplotype diversity, due to the lack of representation by available sequences (Appendix S10). No haplotypes were shared by species among the specimens studied.

### 3.5 Extension of DNA barcode library and mitochondrial genome

The 24 complete mitochondrial genome sequences produced in this study belong to three species, *C. castaneus* (*N* = 19), *C. stigmaticus* (*N* = 4), and *C. randalli* (*N* = 1). These represent the first complete mitogenome sequences for the genus *Cirripectes*. The complete mitogenomes of *C. castaneus*, *C. randalli*, and *C. stigmaticus* are circular with sizes ranging from 16,476 to 16,512 bp (43.3% GC mean), 16,482 bp (43.6% GC), and 16,482 to 16,532 bp (43.8 % GC mean, Appendix S11.1), respectively. More detailed information is provided in Appendix S11.

### 3.6 Sliding window analyses

**FIGURE 3:**
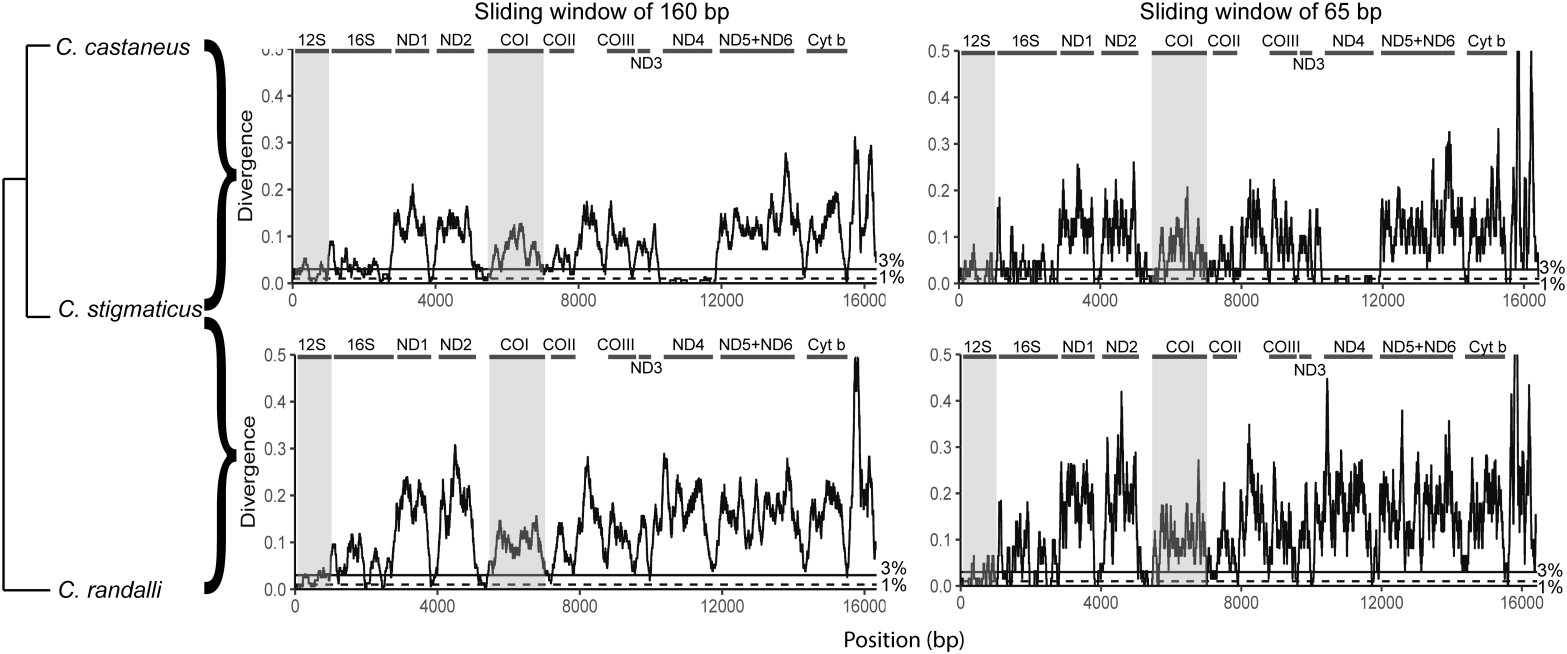
Sliding window analyses of the alignment between *C. castaneus* and *C. stigmaticus* (up) and *C. stigmaticus* and *C. randalli* (bottom). Distances are in K2P (Kimura, 1980). The horizontal lines show divergence threshold: dashed: 1%, full: 3%. Gene boundaries are indicated. Gray box indicates the regions of 12S and COI genes usually targeted for amplification. To improve visibility, values greater than 0.5 (only present in the control region) were cut.

Two pairwise comparisons between the most closely related species pair (*C. castaneus* with *C. stigmaticus*) and the more distant one (*C. stigmaticus* with *C. randalli*), were chosen for sliding window analysis. The distribution of divergent sites is shown in Figure 3. The alignment of the complete mitogenome of *C. castaneus* (Accession number OP749996) with that of *C. stigmaticus* (OP575312) was 16,485 bp long and contained 1162 variable characters. The uncorrected pairwise distance overall between this pair of taxa was 7.1% (p‐distance; Srivathsan & Meier, 2012). The alignment of the complete mitogenome of *C. stigmaticus* with that of *C. randalli* (OP749983) was 16,489 bp long and contained 1795 variable characters corresponding to an uncorrected pairwise distance of 10.9% (p‐distance). The divergence between species was calculated specifically for four fragments amplified by several commonly used primers pairs (Table 3). The PCR in silico failed for the MiFish primers pair (Miya et al., 2015) as no binding site was found for the reverse primer. The complete COI fragment, partial COI from Geller et al. (2013) (jgLCO1490‐jdHCO2198), and Teleo primers were efficiently amplified in silico and discriminated species with a divergence threshold of 3% (in p or K2P distances: minimal differences between models which rarely affect the identification success rates [Collins et al., 2012]). However, prior to the novel sequences presented here, only one reference sequence was available for the 12S fragment targeted by the Teleo primers, therefore only the COI barcodes could be used for species identification. Moreover, this sequence attributed to *C. polyzona* [LC278140.2] was identical to our *C. castaneus* sequences.

**TABLE 3:** Nucleotide divergence between *C. castaneus* and *C. stigmaticus*, and between *C. stigmaticus* and *C. randalli*.

Primers	Reference	Length (bp)	% div (p‐distance)	
			*C. castaneus* vs. *C. stigmaticus*	*C. stigmaticus* vs. *C. randalli*
Complete COI	–	1560	6.3% (99 bp)	9% (140 bp)
jgLCO1490‐jdHCO2198 (COI)	Geller et al. (2013)	658	4.9% (32 bp)	8.8% (58 bp)
MiFish (12S)	Miya et al. (2015)	163–185	4.5%^a^ (7 bp)	2.5%^a^ (4 bp)
Teleo (12S)	Valentini et al. (2016)	63	6.3% (4 bp)	6.3% (4 bp)

^a^ PCR in silico failed to find the biding site for the reverse primer of MiFish. Divergence was estimated from the end of the forward primer to the 160 following pb.

### 3.7 *Cirripectes* species of Reunion and Rodrigues

Following the results of the delimitation analyses, from the 34 new COI sequences generated in this study, 28 were assigned to *C. castaneus*, 4 to *C. stigmaticus*, and two to *C. randalli*. Among the 26 specimens sequenced for rhodopsin, 22 were assigned to *C. castaneus*, 3 to *C. stigmaticus*, and one to *C. randalli*. Of the 29 specimens sampled in Reunion, 27 were assigned to *C. castaneus*, one to *C. stigmaticus*, and one to *C. randalli*. Of the 5 *Cirripectes* samples from Rodrigues, one was assigned to *C. castaneus*, three to *C. stigmaticus*, and one to *C. randalli*. Photographs of the three species are presented in Appendix S12. Each species has a distinct color pattern: *C. castaneus* has orange to red vertical bars on the head and anterior body half, and orange‐red colored upper and lower caudal fin rays, *C. randalli* has orange‐red dots on the head and body, and *C. stigmaticus* has orange‐red vertical bars on the head and dots on the anterior body half (Appendix S12). Their sizes varied from 29 to 67 mm with a median size of 37 mm for *C. castaneus*, 41–44 mm for *C. randalli*, and 34–47 mm for *C. stigmaticus* (Appendix S2).

*Cirripectes* species previously reported from Reunion and Rodrigues are shown in Table 4, as based on visual surveys (Fricke et al., 2009; Letourneur et al., 2004), morphological (Fricke, 1999; Heemstra et al., 2004; Williams, 1988), and molecular studies (Collet et al., 2017; Hubert et al., 2015). Some of the original reference sequences for *Cirripectes* samples from Reunion were misidentified: it turned out that some *C. stigmaticus* were *C. castaneus* (BOLD:AAE2835) and all *C. castaneus* were *C. randalli* (BOLD:AAU0601; Hoban & Williams, 2020). Thus, *C. stigmaticus* (BOLD:AAE2834) was not genetically identified from Reunion prior to the present study (Table 4).

**TABLE 4:** *Cirripectes* species reported from Mascarene Islands.

*C. auritus*					*C. castaneus*					*C. filamentosus*					*C. gilberti*					*C. polyzona*					*C. quagga*					*C. stigmaticus*					*C. randalli*					Meth.	Studies
Re	Ma	Ro	Ca	Ag	Re	Ma	Ro	Ca	Ag	Re	Ma	Ro	Ca	Ag	Re	Ma	Ro	Ca	Ag	Re	Ma	Ro	Ca	Ag	Re	Ma	Ro	Ca	Ag	Re	Ma	Ro	Ca	Ag	Re	Ma	Ro	Ca	Ag		
x	X	x	x	x	✔	✔	x	✔	✔	x	x	x	x	✔	x	x	x	✔	x	x	x	x	X	x	✔	x	x	✔	✔	x	✔	x	✔	x	x	✔	x	✔	x	M	Williams (1988)
	✔																																							M	Debelius (1993)
x	✔	x	x	x	✔	✔	x	✔	✔	x	x	x	x	x	x	x	x	x	x	✔	✔	x	X	x	✔	✔	x	✔	✔	x	✔	✔	✔	x	✔	✔	x	✔	x	M	Fricke (1999)
x	–	–	–	–	✔	–	–	–	–	x	–	–	–	–	x	–	–	–	–	✔	–	–	–	–	✔	–	–	–	–	✔	–	–	–	–	✔	–	–	–	–	V	Letourneur et al. (2004)
–	–	x	–	–	–	–	✔	–	–	–	–	✔	–	–	–	x	✔	–	–	–	–	x	–	–	–	–	x	–	–	–	–	✔	–	–	–	–	x	–	–	M	Heemstra et al. (2004)
x	–	–	–	–	✔	–	–	–	–	x	–	–	–	–	x	–	–	–	–	✔	–	–	–	–	✔	–	–	–	–	✔	–	–	–	–	✔	–	–	–	–	V	Fricke et al. (2009)
x	–	–	–	–	^a^	–	–	–	–	x	–	–	–	–	x	–	–	–	–	x	–	–	–	–	x	–	–	–	–	^a^	–	–	–	–	^b^	–	–	–	–	G	Hubert et al. (2015)
x	–	–	–	–	^a^	–	–	–	–	x	–	–	–	–	x	–	–	–	–	x	–	–	–	–	x	–	–	–	–	^a^	–	–	–	–	^b^	–	–	–	–	G	Collet et al. (2017)
x	–	–	–	–	✔	–	–	–	–	✔	–	–	–	–	x	–	–	–	–	✔	–	–	–	–	✔	–	–	–	–	✔	–	–	–	–	✔	–	–	–	–	V	Wickel et al. (2020)
x	–	x	–	–	✔	–	✔	–	–	x	–	x	–	–	x	x	x	–	–	x	–	x	–	–	x	–	x	–	–	✔	–	✔	–	–	✔	–	✔	–	–	G	Present study

*Note*: Dark gray for the present, light gray for not observed, and white for not evaluated. Methods of identification were synthetized as follows (Meth.): M for morphometric analysis, V for visual survey, and G for molecular identification.

Abbreviations: Ag: Agalega Islands; Ca: Cargados Carajos Shoals (Saint Brandon); Ma: Mauritius; Re: Reunion; Ro: Rodrigues.

^a^ Misidentification between *C. stigmaticus* and *C. castaneus*.

^b^ Misidentification between *C. randalli* and *C. castaneus*.

## 4 DISCUSSION

### 4.1 Phylogeny of *Cirripectes*

Phylogenetic trees constructed on the COI gene were the most informative since the availability of the sequences of the alternative genes for the other species and localities was limited. The present study supplements the range of available markers for future analyses. Phylogenetic trees corroborated the monophyly of the genus *Cirripectes*. Despite some differences in internal branching order, the COI trees were generally congruent with the morphological phylogeny established by Williams (1988) and recent single marker phylogenies also based on the COI gene (Delrieu‐Trottin et al., 2018; Hoban & Williams, 2020). The differences observed between our results and phylogenies produced by Hoban and Williams (2020) were among the short branches and poorly supported clades in both studies. The low resolution among these branches is probably due to multiple rapid divergence events with little time to accumulate shared mutations, even in a fast‐evolving marker (Avise, 2009; Douzery, 2010). The use of additional genetic markers with greater variability, and a better taxon coverage for the genetic markers available, may resolve the remaining topological uncertainties or confirm the existence of fast diversification events.

### 4.2 Molecular delimitation of *Cirripectes*

The number of clusters recovered by molecular species delineation approaches using the COI gene depends on the method used. In our case, the number of recovered clusters varied from 16 to 19. The ASAP delimitation approach generated less clusters than the tree‐based methods GMYC and mPTP, which is consistent with the literature (Dvořák et al., 2022; Kekkonen & Hebert, 2014; Puillandre et al., 2021). These results highlight the importance of performing multiple molecular approaches to determine the congruent clusters. However, using multiple molecular markers (ideally from mitochondrial and nuclear DNA) combined with geographically wide sampling is needed to resolve remaining uncertainties. In addition, independent evidence, such as ecological data or morphological characters must be examined on all the specimens for integrative taxonomy and validation of the molecular species hypothesis (Puillandre et al., 2021). Our phylogeny and delimitation analyses also support the previous molecular distinction between *Cirripectes alboapicalis*, *Cirripectes patuki* sensu Delrieu‐Trottin et al. (2018), and a new species (Delrieu‐Trottin et al., 2018). Therefore, the use of the COI gene alone as a barcode could be sufficient for *Cirripectes* identification.

### 4.3 *Cirripectes* in the Mascarene Islands

The type localities of two *Cirripectes* species are in the Mascarene Islands. The holotype of *Salarias castaneus* Valenciennes in Cuvier and Valenciennes (1836) was collected at Isle de France (Mauritius). This species was later assigned to *Cirripectes* and, according to Williams (1988), several times erroneously synonymized with *C. variolosus* (e.g., Smith, 1959). The holotype of *C. randalli* Williams, 1988 originates from Cargados Carajos Shoals and paratypes come from Mauritius. Moreover, one paratype of *C. gilberti* Williams, 1988 is from Agalega. However, to date, few studies comprehensively assessed the diversity and distribution of *Cirripectes* species in the Mascarene archipelago.

*Cirripectes* occurrence at Agalega, Cargados Carajos Shoals, Mauritius, and Reunion was first described by Williams in Williams, 1988 (Table 3). *C. castaneus* and *C. quagga* were listed for Reunion, with the checklist subsequently completed based on visual records by Letourneur (1992) with *C. polyzona* by Fricke (1999) with *C. stigmaticus* and *C. randalli*, and by Wickel et al. (2020) with *C. filamentosus*. Subsequent checklists were based on the previous ones, sometimes without the addition of new *Cirripectes* species (Fricke et al., 2009; Letourneur et al., 2004). Heemstra et al. (2004) provided a preliminary fish list for Rodrigues and referred to the earlier studies of Gunther (1879), de Baissac (1968) and Fricke (1999) as problematic, due to misidentifications and undocumented sight records (Heemstra et al., 2004). The *Cirripectes* sequences found in public databases were produced by studies not focused on *Cirripectes* but on taxonomically broad barcoding efforts of Indo‐Pacific coral‐reef fishes (Hubert et al., 2012) and post‐larvae (Collet et al., 2017).

Both on Reunion and Rodrigues, *C. castaneus*, *C. randalli*, and *C. stigmaticus* were collected using ARMS. This means that ARMS sampled three out of the 6 *Cirripectes* species listed for Reunion and three out of four species listed for Rodrigues (Table 4). These results were in agreement with the previous molecular studies available for Reunion, which reported *C. castaneus* and *C. randalli* (Collet et al., 2017; Hubert et al., 2015). To the best of our knowledge, we provide the first record of *C. randalli* for Rodrigues and the first sequence of *C. stigmaticus* for Reunion.

In spite of the extensive sampling using ARMS at Reunion (46 ARMS deployed at 10 sites in the course of 7 years), the absence of the other three species recorded for the island by visual censuses (*C. filamentosus*, *C. polyzona*, and *C. quagga*) can be explained by several hypotheses: (i) their habitats were not sampled, (ii) ARMS are not suitable to sample these species, (iii) these species are not present on the island, or (iv) were not present at the time of sampling.

The first possibility is that the sampling sites did not include the habitats of the species not sampled. Indeed, we sampled only on the top of spurs at 10–12 m depth, whereas some species (*C. polyzona* and *C. quagga*) are reported to prefer algal ridges and crests in the surf zone (Williams, 1988). Also, the public sequences of *C. randalli* were from specimens collected in tide pools or on shallow reef flats (JHB, pers. comm.). The difference in the habitat sampling may explain the low number of *C. randalli* recovered using ARMS on outer reef slopes.

The second explanation pertains to the sampling ability of ARMS for certain species. The ARMS are deployed on the reef surface, whereas other methods, such as rotenone or clove oil sampling, can potentially access cryptic species that live deeper within the reef matrix. In spite of earlier rotenone sampling at Reunion at various locations and depths (Hubert et al., 2012), only *C. castaneus* and *C. randalli* were recovered. In contrast, ARMS sampling allowed recovering an additional species, *C. stigmaticus*.

The third explanation rests on the hypothesis that the other three species do not occur on Reunion. From the first observation of *Cirripectes*, it has been reported that the species can be highly variable, leading to numerous misidentifications, even when morphology could be studied in detail (Smith, 1959; Williams, 1988). Thus, visual surveys without detailed examination of the morphological characters may not permit to reliably identify all *Cirripectes* species. The species *C. filamentosus*, *C. polyzona*, and *C. quagga* listed for Reunion based exclusively on visual records might correspond to erroneous identifications. Ideally, the visual records of the “missing” species need to be confirmed by genetic analyses.

Fourth, *Cirripectes*, like other cryptobenthic reef fishes, are considered to have an abundant larval supply (Brandl et al., 2019). It is therefore surprising that the “missing” three species have not been recovered during several years of sampling of post‐larvae at Reunion (Adeline Collet, pers. comm.). Moreover, the ARMS sampling were carried out over several years and at different times of the year, including hot and cool seasons. In view of this extensive and diverse sampling, it is unlikely that the three species were missed due to a seasonal effect, and this further supports the hypothesis that these species may in fact not be present at Reunion. Moreover, *C. castaneus* collected with ARMS had smaller sizes (median of 37 mm) than rotenone‐collected specimens from shallow reef flats (median of 71 mm; JHB, pers. comm.), suggesting that ARMS provide habitat for juvenile individuals following recruitment.

Molecular studies are not limited by morphological identification and allow reliable identification of specimens and matching among studies. For this reason, we can confirm with certitude the presence of *C. castaneus*, *C. stigmaticus*, and *C. randalli* in Reunion and Rodrigues. In the same way the COI marker is highly useful for *Cirripectes* identification, the mitochondrial dataset provided here should allow the detection of the three 'missing' species with an eDNA metabarcoding study and resolve the uncertainty about their occurrence in the Mascarene.

### 4.4 Geographical ranges of Mascarene *Cirripectes*

The geographic ranges of the three *Cirripectes* species that we collected in the Mascarene Islands, *C. castaneus*, *C. stigmaticus*, and *C. randalli*, show different patterns. *C. randalli* was reported as an endemic species of the Mascarene Islands and listed in Mauritius, Cargados Carajos Shoals, and Reunion (Fricke, 1999; Williams, 1988). The presence of *C. randalli* in Reunion confirmed in this study is consistent with the literature and previous molecular studies. To the best of our knowledge, this is the first report of *C. randalli* from Rodrigues, which is congruent with its known geographical range. *C. castaneus* and *C. stigmaticus* are both reported to have an Indo‐Pacific wide distribution. Our analyses confirm this and showed a lack of divergence and genetic structuration among sequences from various locations. Moreover, for both species, several haplotypes were shared among distant localities such as Seychelles, Reunion, and New Caledonia for *C. castaneus*, and the Mascarene Islands to Fiji and Wallis & Futuna for *C. stigmaticus*. Surprisingly, for other species reported to have very wide distributions (*C. auritus*, *C. filamentosus*, *C. polyzona*, *C. quagga*, and *C. variolosus*) no sequences have been recovered from geographically distant sites. These results may be an artifact of uneven sampling efforts among localities. Alternatively, given the recent highlight of cryptic species with small endemism areas in the genus (Delrieu‐Trottin et al., 2018; Hoban & Williams, 2020), this could be the outcome of multiple cryptic lineages in the genus and in need of further investigation. Indeed, the presence of cryptic species could have implications for understanding mechanisms driving biodiversity patterns (Eme et al., 2018). Cryptic species have distinct evolutionary patterns and, in some cases, a restricted geographical range with specialized behavior or higher threat susceptibility. Therefore, the detection of cryptic species is crucial for the conservation and/or management of marine biodiversity (Bickford et al., 2007).

### 4.5 Extending and using DNA databases for specimen identification

It is of utmost importance to share molecular datasets that can be cross‐checked and used openly for taxonomic or identification purposes. Private datasets limit error detection and correction, and the comparability of many current metabarcoding studies is limited because of the inaccessibility of the datasets used for taxon assignment. The newly added sequences expand the reference public DNA database of *Cirripectes* to 85 individual sequences. The new sequences are all attached to a specimen deposited in a registered collection and correspond therefore to genseq‐3 (collection‐vouchered non‐types) according to the nomenclature of Chakrabarty et al. (2013). Of these, 24 represent the first mitogenome sequences for the genus and these three species. These additions will support future studies using mitochondrial markers other than COI for specimen identification or phylogenetic reconstruction. Available complete mitogenomes enable the selection of the most appropriate marker to respond to the objectives of such studies. Likewise, we deposited the first *Cirripectes* sequences from Rodrigues for three species and added 23 sequences to the 14 *Cirripectes* sequences from Reunion. Finally, this study added 45 new sequences for the rhodopsin gene (27 specimens with 18 heterozygotes) to the existing 12 public sequences (8 specimens with 3 heterozygotes). There are still very few *Cirripectes* sequences for nuclear markers, and the rhodopsin gene was one of the most represented with eight sequences.

Sequences previously deposited in the BOLD and GenBank databases and previous studies allowed us to assign the specimens we collected to two taxonomic names. Further analyses revealed the existence of misidentified specimens in these databases. Some of these misidentifications have already been corrected and published but were not yet corrected in the BOLD database (BOLD:ADB2362 probably do not correspond to *Cirripectes* specimens in Chu et al., 2019; BOLD:AAU0601 is *C. randalli* in Hoban & Williams, 2020). The presence and problem of misidentified specimens in reference databases are well documented, even for Indo‐Pacific fishes (Leis, 2015; Pentinsaari et al., 2020). In fact, even when authors are aware of this problem, the wrong assignation may occur. Indeed, if a specimen is assigned to a BOLD BIN that contains specimens assigned to different species with no obvious errors about the geographic distribution, deciding which name is correct is problematic. Similar problems arise when a species corresponds to several BOLD BINs. To resolve these uncertainties, a new morphological examination of the specimens must be performed and, if needed, an investigation of phylogenetic relationships, possibly with added specimens and species (Ward et al., 2009).

### 4.6 Primer selection and perspectives

Among the technical issues in molecular ecology, the choice of primer for PCR amplification is one of the most important factors affecting the probability of species detection. The eDNA approach led to the design of primers to respond to the new constraints, such as short ID sequences (<200 bp) to improve PCR success with degraded eDNA (Bylemans et al., 2018; Freeland, 2017). In the case of *Cirripectes*, two widely used primer pairs targeting the 12S were tested for comparison with results from the longer COI barcode. Unlike the proprietary Teleo primers (Valentini et al., 2016), the MiFish primer set failed to amplify species *in sillico*, which contrasts with the results of Zhang et al. (2020) that showed that the latter primers had a larger detection range than the Teleo primer set. The genome areas targeted by COI and Teleo primers had a divergence >3% and thus allow automated species identification using the commonly used threshold for discriminating potential species. However, for the 12S targeted by the Teleo primers, only one sequence was available as a reference for this genus before our study, therefore, only the COI barcodes could truly be used for species identification.

In the future, primer limitations for eDNA surveys will be overcome with the development of shotgun sequencing for the eDNA, by direct sequencing of total eDNA and bypassing the PCR limitations associated with metabarcoding to provide insights into community composition (Taberlet et al., 2012; Tringe & Rubin, 2005). Currently, this approach is still limited by the completeness of the reference databases, in terms of species diversity (as barcoding) and in terms of reference genomes coverage (e.g., complete mitochondrial DNA).

Finally, multiplexed NGS sequencing of long amplicons produced cost‐efficient complete mitogenomes, providing sequences for markers with greater variability than available previously. In the case of *Cirripectes*, ND1 and ND2 may be used instead of COI to resolve the remaining uncertainties. Moreover, using reads from NGS sequencing enables easy differentiation of alleles for nuclear markers.

## 5 CONCLUSION

In this study, the geographic distribution and species relationships within genus *Cirripectes* were examined. The major conclusions are summarized below:

1. At Reunion, numerous replicates of ARMS allowed sampling of one additional species in comparison with rotenone or light‐trap captures, repeated in space and time.
2. The presence of *C. castaneus*, *C. randalli*, and *C. stigmaticus* in Reunion and Rodrigues was confirmed. However, the species *C. filamentosus*, *C. polyzona*, and *C. quagga*, listed as present in Reunion based on visual identifications, were not found and may correspond to erroneous identifications.
3. The generated data contribute to filling the gaps in taxonomic and molecular knowledge of reef cryptobiome for the South‐West Indian Ocean, and the first complete mitogenomes of three *Cirripectes* species are provided.
4. Both the COI gene and the target of the eDNA Teleo primer set have interspecific divergence >3% and allow species identification for Mascarene *Cirripectes*.
5. COI sequences are not sufficient to clearly resolve the relationships among *Cirripectes* species.

## AUTHOR CONTRIBUTIONS

**Marion Couëdel:** Conceptualization (equal); data curation (lead); formal analysis (lead); investigation (lead); project administration (equal); visualization (lead); writing – original draft (lead); writing – review and editing (lead). **Agnes Dettai:** Conceptualization (equal); formal analysis (supporting); funding acquisition (supporting); investigation (equal); methodology (lead); project administration (equal); validation (lead); writing – review and editing (equal). **Mireille M. M. Guillaume:** Conceptualization (equal); investigation (equal); project administration (equal); writing – review and editing (equal). **Fleur Bruggemann:** Investigation (equal). **Sophie Bureau:** Investigation (equal). **Baptiste Frattini:** Investigation (equal). **Amélie Verde Ferreira:** Investigation (equal). **Jean‐Lindsay Azie:** Investigation (equal). **J. Henrich Bruggemann:** Conceptualization (lead); funding acquisition (lead); investigation (equal); project administration (lead); writing – review and editing (equal).

## CONFLICT OF INTEREST STATEMENT

The authors state that they have no conflicting interests.

## Supporting information


Appendix S1–S12

Data S1

Data S2

Data S3

Data S4

## Data Availability

All sequences produced in this study were deposited on GenBank and Bold databases. Accession numbers are provided in Appendix S2 and at the following DOI: 10.5281/zenodo.7599910. R code to reproduce findings is available on MC (Mcouedel) github.
