# Supplementary material for: New insights into the diversity of cryptobenthic Cirripectes blennies in the Mascarene Archipelago sampled using Autonomous Reef Monitoring Structures (ARMS)
Source: Ecol Evol. 2023 Mar 16;13(3):e9850. doi: 10.1002/ece3.9850 (PMC10019914; doi:10.1002/ece3.9850)

## Appendices

Appendix 1: ARMS deployments and *Cirripectes* spp collected. * designates sites located inside no-fishing zones.

| **Island** | **Site** | **Soak time (yrs)** | **Deployment** | **Recovery** | **ARMS recovered** | **Latitude**  **(N)** | **Longitude (E)** | ***C. castaneus*** | ***C. randalli*** | ***C. stigmaticus*** | **Unidentified** | **Total** |
| --- | --- | --- | --- | --- | --- | --- | --- | --- | --- | --- | --- | --- |
| **Reunion** | RUNA2* | 0.6 | 08/09/2014 | 22-24/04/2015 | 3 | -21.103883 | 55.235917 | 2 |  |  |  | 2 |
|  | RUNA2* | 4.5 | 08/04/2014 | 17/12/2018 | 2 | -21.103883 | 55.235917 | 1 |  |  |  | 1 |
|  | GILA1* | 4 | 01/11/2014 | 12/2018 | 2 | -21.077183 | 55.215967 | 2 |  |  |  | 2 |
|  | RUNA1 | 2 | 23/01/2019 | 14-17/01/2021 | 3 | -21.02060 | 55.22983 | 1 |  |  |  | 1 |
|  | RUNA2* | 2 | 17/12/2018 | 14/12/2020  13/01/2021 | 3 | -21.10401 | 55.23598 | 5 | 1 | 1 | 1 | 8 |
|  | RUNA3* | 2 | 23/01/2019 | 15-17/01/2021 | 3 | -21.10411 | 55.23604 | 7 |  |  |  | 7 |
|  | RUNA4* | 2 | 18/12/2018 | 19/12/2020  08/01/2021 | 3 | -21.17256 | 55.28228 |  |  |  |  |  |
|  | RUNA5* | 2 | 18/12/2018 | 18/12/2020  15/01/2021 | 3 | -21.17352 | 55.28249 | 3 |  |  |  | 3 |
|  | RUNA6 | 2 | 23/01/2019 | 14-19/01/2021 | 3 | -21.191040 | 55.282821 |  |  |  |  |  |
|  | RUNA7* | 2 | 25/01/2019 | 19-20/01/2021 | 3 | -21.269565 | 55.327891 |  |  |  | 1 | 1 |
|  | RUNA8 | 2 | 25/01/2019 | 18/01/2021  08/02/2021 | 3 | -21.35209 | 55.48074 | 1 |  |  | 1 | 2 |
|  | RUNA9 | 2 | 25/01/2019 | 20/01/2021  09/02/2021 | 3 | -21.37236 | 55.54692 | 1 |  |  | 1 | 2 |
|  | RUNA2* | 0.5 | 25/02/2020 | 27-31/08/2020 | 3 | -21.10401 | 55.23598 | 1 |  |  |  | 1 |
|  | RUNA2* | 1 | 25/02/2020 | 20-22/02/2021 | 3 | -21.10401 | 55.23598 |  |  |  | 1 | 1 |
|  | RUNA2* | 0.5 | 31/08/2020 | 19/02/2021 | 3 | -21.10401 | 55.23598 | 3 |  |  |  | 3 |
|  | RUNA2* | 1 | 31/08/2020 | 26-31/08/2021 | 3 | -21.10401 | 55.23598 |  |  |  |  |  |
| **Rodrigues** | RODA1 | 2 | 08/12/2015 | 11-12/01/2017 | 3 | -19.66878 | 63.47387 |  |  |  |  |  |
|  | RODA2 | 2 | 10/12/2015 | 13-16/01/2017 | 3 | -19.65262 | 63.40073 | 1 | 1 | 2 |  | 4 |
|  | RODA3 | 2 | 11/12/2015 | 16-17/01/2017 | 2 | -19.65397 | 63.41513 |  |  | 1 |  | 1 |
| **Total** |  |  |  |  | 54 |  |  | 28 | 2 | 4 | 5 | 39 |

Appendix 2: Sequences generated during this study and specimen total length

|  |  | **GenBank Accession Number** | | |  |  |  |  | |  |  |  |
| --- | --- | --- | --- | --- | --- | --- | --- | --- | --- | --- | --- | --- |
| **Field ID** | **Species** | **Complete mitogenome** | **COI** | **Rho** | **BOLD** | **Collection Date** | **Country** | **Site** | **ARMS** | **Soak time (yrs)** | **Season** | **Length (mm)** |
| RUNA_0272 | *castaneus* | OP820448 |  |  | IOACT113-22 | 2018 | Reunion | GILA1 | GILA1A | 4 | Hot | 35 |
| RUNA_0370 | *castaneus* | OP820446 |  |  | IOACT112-22 | 2018 | Reunion | GILA1 | GILA1B | 4 | Hot | 39 |
| RUNA_2168 | *castaneus* | OP749990 |  | OP776358 / OP776359 | IOACT040-21 | 2020 | Reunion | RUNA1 | RUNA1B | 2 | Hot | 36 |
| ORCIE1027 | *castaneus* | OP820445 |  |  | IOACT115-22 | 2018 | Reunion | RUNA2 | SALA1A | 4 | Hot | 62 |
| ORCIE1125 | *castaneus* | OP820447 |  |  | IOACT116-22 | 2018 | Reunion | RUNA2 | SALA1B | 4 | Hot | 56 |
| RUNA_0022 | *castaneus* | OP820444 |  |  | IOACT114-22 | 2018 | Reunion | RUNA2 | SALA1D | 4 | Hot | 35 |
| RUNA_0737 | *castaneus* | OP749987 |  | OP776352 / OP776353 | IOACT025-21 | 2020 | Reunion | RUNA2 | CINA1C | 0,5 | Cool | 38 |
| RUNA_2899 | *castaneus* | OP749997 |  |  | IOACT053-21 | 2020 | Reunion | RUNA2 | RUNA2A | 2 | Hot | NA |
| RUNA_3154 | *castaneus* | OP749998 |  | OP776378 / OP776379 | IOACT057-21 | 2021 | Reunion | RUNA2 | RUNA2B | 2 | Hot | 67 |
| RUNA_3155 | *castaneus* | OP749999 |  | OP776380 / OP776381 | IOACT058-21 | 2021 | Reunion | RUNA2 | RUNA2B | 2 | Hot | 30 |
| RUNA_3176 | *randalli* | OP749984* |  | OP776382 / OP776383 | IOACT059-21 | 2021 | Reunion | RUNA2 | RUNA2B | 2 | Hot | 44 |
| RUNA_3214 | *castaneus* | OP750000 |  | OP776384 / OP776385 | IOACT061-21 | 2021 | Reunion | RUNA2 | RUNA2C | 2 | Hot | 29 |
| RUNA_3262 | *stigmaticus* | OP575312 |  |  | IOACT088-22 | 2021 | Reunion | RUNA2 | RUNA2C | 2 | Hot | NA |
| RUNA_3895 | *castaneus* | OP750001 |  | OP776388 / OP776389 | IOACT075-21 | 2021 | Reunion | RUNA2 | CINA3B | 0,5 | Hot | 32 |
| RUNA_4001 | *castaneus* | OP750002 |  | OP776390 / OP776391 | IOACT078-21 | 2021 | Reunion | RUNA2 | CINA3C | 0,5 | Hot | 47 |
| RUNA_4002 | *castaneus* | OP750003 |  | OP776392 / OP776393 | IOACT079-21 | 2021 | Reunion | RUNA2 | CINA3C | 0,5 | Hot | 34 |
| RUNA3256 | *castaneus* | OP749986 |  | OP776386 / OP776387 | IOACT063-21 | 2021 | Reunion | RUNA2 | RUNA2C | 2 | Hot | 54 |
| RUNA_2344 | *castaneus* | OP749991 |  | OP776360 / OP776361 | IOACT043-21 | 2020 | Reunion | RUNA3 | RUNA3A | 2 | Hot | 48 |
| RUNA_2346 | *castaneus* | OP749992 |  | OP776362 / OP776363 | IOACT087-22 | 2020 | Reunion | RUNA3 | RUNA3A | 2 | Hot | 29 |
| RUNA_2347 | *castaneus* | OP749993 |  | OP776364 / OP776365 | IOACT044-21 | 2020 | Reunion | RUNA3 | RUNA3A | 2 | Hot | 34 |
| RUNA_2348 | *castaneus* |  | OP787979 | OP776366 / OP776367 | IOACT086-22 | 2020 | Reunion | RUNA3 | RUNA3A | 2 | Hot | 42 |
| RUNA_2379 | *castaneus* | OP749994 |  | OP776368 / OP776369 | IOACT045-21 | 2020 | Reunion | RUNA3 | RUNA3B | 2 | Hot | 59 |
| RUNA_2449 | *castaneus* | OP749995 |  | OP776370 / OP776371 | IOACT047-21 | 2020 | Reunion | RUNA3 | RUNA3C | 2 | Hot | 30 |
| RUNA_2450 | *castaneus* | OP749996 |  | OP776372 / OP776373 | IOACT048-21 | 2020 | Reunion | RUNA3 | RUNA3C | 2 | Hot | 30 |
| RUNA_2717 | *castaneus* |  | OP787978 | OP776374 / OP776375 | IOACT090-22 | 2020 | Reunion | RUNA5 | RUNA5A | 2 | Hot | NA |
| RUNA_2718 | *castaneus* |  | OP787977 | OP776376 / OP776377 | IOACT089-22 | 2020 | Reunion | RUNA5 | RUNA5A | 2 | Hot | 33 |
| RUNA_2719 | *castaneus* | OP820449 |  |  | IOACT084-22 | 2020 | Reunion | RUNA5 | RUNA5A | 2 | Hot | 31 |
| RUNA_0854 | *castaneus* | OP749988 |  | OP776354 / OP776355 | IOACT026-21 | 2021 | Reunion | RUNA8 | RUNA8A | 2 | Hot | 67 |
| RUNA_1208 | *castaneus* | OP749989 |  | OP776356 / OP776357 | IOACT034-21 | 2021 | Reunion | RUNA9 | RUNA9A | 2 | Hot | 44 |
| ORCIE1808 | *castaneus* | OP749985 |  | OP776344 / OP776345 | IOACT091-22 | 2017 | Rodrigues | RODA2 | RODA2A | 2 | Hot | 38 |
| ORCIE1889 | *stigmaticus* | OP575310 |  | OP776346 / OP776347 | IOACT092-22 | 2017 | Rodrigues | RODA2 | RODA2B | 2 | Hot | 46 |
| ORCIE1890 | *stigmaticus* | OP575311 |  | OP776348 / OP776349 | IOACT016-21 | 2017 | Rodrigues | RODA2 | RODA2B | 2 | Hot | 34 |
| ORCIE1990 | *randalli* | OP749983 |  | OP776350 / OP776351 | IOACT021-21 | 2017 | Rodrigues | RODA2 | RODA2C | 2 | Hot | 41 |
| 1583 | *stigmaticus* | OP575309 |  | OP776342 / OP776343 | IOACT001-21 | 2017 | Rodrigues | RODA3 | RODA3A | 2 | Hot | 47 |

Appendix 3: Outgroups for each dataset.

| Species | GenBank Accession Number | | |
| --- | --- | --- | --- |
|  | **COI** | **Rho** | **Complete mitogenome (mt)** |
| *Ophioblennius macclurei* | HQ168577.1 | HQ168928 .1 |  |
| *Omobranchus elegans* | KT284893.1 |  | KT284893.1 |
| *Omobranchus obliquus* |  | HQ168927.1 |  |
| *Petroscirtes breviceps* | NC_004411.1 | KF265117.1 | NC_004411.1 |
| *Salarias fasciatus* | NC_004412.1 | HQ168942.1 | NC_004412.1 |
| *Ecsenius bicolor* | NC_028295.1 |  | NC_028295.1 |

Appendix 4: Proportions of variable and parsimony informative characters and best nucleotide substitution model for each partition of the datasets.

| Dataset | Partition | Codon position | #seq | Length (bp) | Variable characters | Parsimony informative characters | Best/fit partitioning scheme |
| --- | --- | --- | --- | --- | --- | --- | --- |
| COI | COI | 1^st^ | 296 | 168 | 20 (11.90%) | 19 (11.31%) | SYM+G4 |
|  |  | 2^nd^ | 296 | 168 | 0 | 0 | F81+F |
|  |  | 3^rd^ | 296 | 168 | 154(91.67%) | 151 (89.88%) | GTR+I+G4 |
|  | COI |  | 292 | 506 | 176 (34.78%) | 170 (33.60%) | GTR+F+I+G4 |
| mt | 12S |  | 24 | 969 | 283 (29.21%) | 152 (15.69%) | GTR+I+G4 |
|  | 16S |  | 24 | 1708 | 675 (39,52%) | 378 (22.13%) | GTR+I+G4 |
|  | CDS | 1^st^ | 24 | 3802 | 1377 (36.22%) | 845 (22.23%) | GTR+I+G4 |
|  |  | 2^nd^ | 24 | 3801 | 1418 (37.31%) | 1020 (26.84%) | GTR+I+G4 |
|  |  | 3^rd^ | 24 | 3801 | 2623 (69,01%) | 1877 (49.38%) | GTR+I+G4 |
| Rho | Rho | 1^st^ | 70 | 245 | 18 (7.35%) | 6 (2.45%) | TVM+F+G4 |
|  |  | 2^nd^ | 70 | 245 | 8 (3.27%) | 4 (1.63%) | F81+F+G4 |
|  |  | 3^rd^ | 70 | 245 | 81 (33.06%) | 39 (15.92%) | GTR+F+G4 |

Appendix 5: Bayesian Inference (left) and Maximum Likelihood (right) trees of concatenated two rRNA (12S and 16S) and coding DNA sequence (CDS) from complete mitogenomes, with the same samples in both trees linked by a line. Intraspecific support values are not shown.


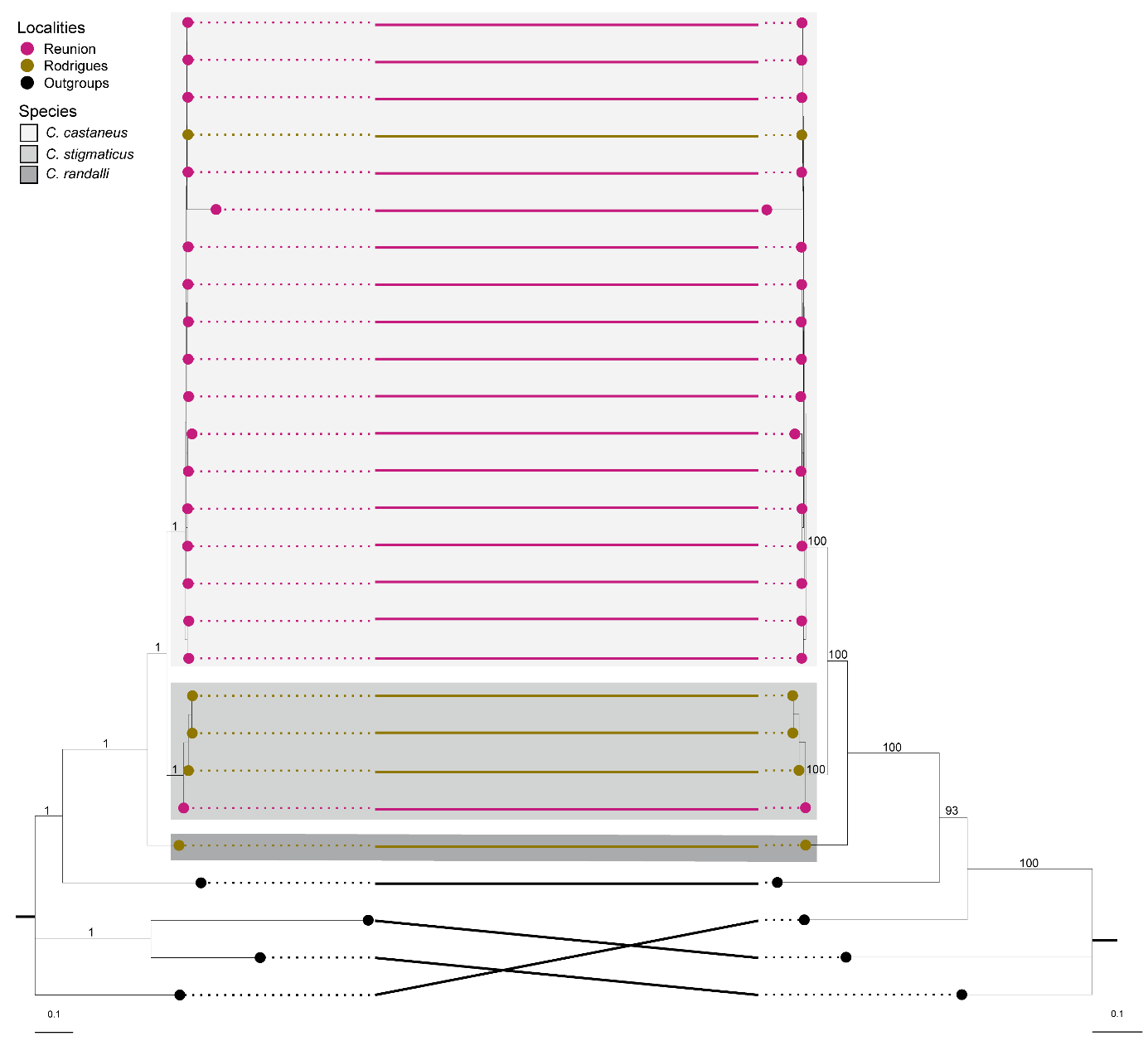


Appendix 6: Population average pairwise difference in bp. Above diagonal: Average number of pairwise differences between species (PiXY); Diagonal elements in grey: Average number of pairwise differences within species (PiX); Below diagonal: Corrected average pairwise difference (PiXY/(PiX+PiY)/2). * for significant p-values.

|  | *C. alboapicalis* | *C. auritus* | *C. castaneus* | *C. chelomatus* | *C. filamentosus* | *C. fuscoguttatus* | *C. jenningsi* | *C. matatakaro* | *C. obscurus* | *C. patuki sensu* Delrieu-Trottin et al. 2018 | *C. polyzona* | *C. quagga* | *C. randalli* | *C.*sp.n. Delrieu-Trottin | *C. stigmaticus* | *C. vanderbilti* | *C. variolosus* |
| --- | --- | --- | --- | --- | --- | --- | --- | --- | --- | --- | --- | --- | --- | --- | --- | --- | --- |
| *C. alboapicalis* | 0 | 72.86 | 62.13* | 75.33 | 79.63 | 75.17* | 63.5 | 69.71* | 42 | 22.2* | 69 | 66.77* | 71* | 47.8* | 71.63* | 77.91* | 69.55* |
| *C. auritus* | 72.57* | 0.57 | 51.69* | 45.67* | 55.38* | 58.67* | 84.36 | 49.47* | 80.14 | 82.86* | 57.29* | 81.08* | 61.54* | 87.94* | 55.66* | 52.67* | 61.56* |
| *C. castaneus* | 60.74* | 50.01* | 2.79 | 46.23* | 50.78* | 56.53* | 69.07* | 43.29* | 61.22 | 67.65* | 46.41* | 62.43* | 48.17* | 67.9* | 27.2* | 46.34* | 32.7* |
| *C. chelomatus* | 75* | 45.05* | 44.51* | 0.67 | 15.38* | 50* | 81.33 | 44.54* | 74.33 | 78.33* | 51 | 69.79* | 56.07* | 82.53* | 49.04* | 47.22* | 58.18* |
| *C. filamentosus* | 77.73* | 53.2* | 47.5* | 13.15* | 3.79 | 54.67* | 81.75* | 45.49* | 79.63 | 80.93* | 53.33* | 71.07* | 59.58* | 79.08* | 52.81* | 45.35* | 60.98* |
| *C. fuscoguttatus* | 74.1* | 57.31* | 54.07* | 48.6* | 51.71* | 2.13 | 81.5* | 47.33* | 72.33 | 77.33* | 52.33* | 71.68* | 53.6* | 80.7* | 48.96* | 47.71* | 62.19* |
| *C. jenningsi* | 60* | 80.57* | 64.17* | 77.5* | 76.36* | 76.93* | 7 | 77.89* | 68.5 | 71.7* | 75.33 | 68.55* | 76.9* | 69.7* | 70.63* | 81.13* | 75.42* |
| *C. matatakaro* | 68.6* | 48.07* | 40.79* | 43.1* | 42.49* | 45.16* | 73.28* | 2.22 | 69.16 | 72.01* | 42.11* | 67.06* | 46.73* | 75.3* | 39.92 | 21.96* | 55.98* |
| *C. obscurus* | 42* | 79.86* | 59.82* | 74* | 77.73* | 71.27* | 65* | 68.05 | 0 | 37.4 | 68* | 66.32* | 71 | 39.2 | 59.5* | 80.29* | 65.25* |
| *C. patuki sensu* Delrieu-Trottin et al. 2018 | 20.7* | 81.07* | 64.76* | 76.5* | 77.53* | 74.77* | 66.7* | 69.4* | 35.9* | 3 | 76.07* | 65.83* | 73.04* | 49.8* | 75.9* | 78.4* | 77.63* |
| *C. polyzona* | 68.33* | 56.33* | 44.35* | 50* | 50.77* | 50.6* | 71.17* | 40.33* | 67.33* | 73.9* | 1.33 | 78.09* | 53.1* | 70.47* | 42.63* | 45.52* | 57.95* |
| *C. quagga* | 66.14* | 80.16* | 60.4* | 68.82* | 68.54* | 69.98* | 64.41* | 65.31* | 65.68* | 63.69* | 76.79* | 1.27 | 72.49* | 68.25* | 71.24* | 74.13* | 68.71* |
| *C. randalli* | 70.12* | 60.38* | 45.89* | 54.86* | 56.8* | 51.66* | 72.52* | 44.75* | 70.12* | 70.66* | 51.56* | 70.98* | 1.76 | 78.9* | 45.93* | 53.28* | 59.2* |
| *C.*sp.n. Delrieu-Trottin et al. 2018 | 47.6* | 87.46* | 66.3* | 82* | 76.98* | 79.43* | 66* | 73.99* | 39* | 48.1* | 69.6* | 67.41* | 77.82* | 0.4 | 72.58* | 74.49* | 75.97* |
| *C. stigmaticus* | 70.66* | 54.41* | 24.84* | 47.74* | 49.96* | 46.93* | 66.16* | 37.84* | 58.54* | 73.44* | 40.99* | 69.64* | 44.08* | 71.41* | 1.93 | 42.95* | 35.69* |
| *C. vanderbilti* | 76.87* | 51.35* | 43.91* | 45.85* | 42.41* | 45.6* | 76.59* | 19.81* | 79.25* | 75.86* | 43.81* | 72.46* | 51.36* | 73.25* | 40.95* | 2.05 | 58.83* |
| *C. variolosus* | 67.44* | 59.16* | 29.2* | 55.73* | 56.98* | 59.02* | 69.81* | 52.76* | 63.14* | 74.02* | 55.17* | 65.96* | 56.21* | 73.66* | 32.61* | 55.68* | 4.22 |

Appendix 7: AMOVA results for among species and within species. s.s.: sum of squares, v.c.: variance components, % var: % of variation, FST: fixation index.

| **AMOVA results for COI** | | | | | |
| --- | --- | --- | --- | --- | --- |
| **Source of variation** | **s.s** | **v.c.** | **% var** | **FST** | **p-value** |
| Among species | 6323.217 | 25.07971 | 94.59 | 0.94589 | 0.000 |
| Within species | 400.317 | 1.43483 | 5.41 |  |  |
| **AMOVA results for rhodopsin** | | | | | |
| **Source of variation** | **s.s** | **v.c.** | **% var** | **FST** | **p-value** |
| Among species | 93.47 | 3.02 | 85.81 | 0.858 | 0.000 |
| Within species | 31.90 | 0.50 | 14.19 |  |  |

Appendix 8: FST matrix between *Cirripectes* species for COI dataset (left) and rhodopsin dataset (right). Blue scale represents FST values (darkest blues for highest FST). Crosses represent single non-significant p-value of the pairwise FST (p-value >0.05).


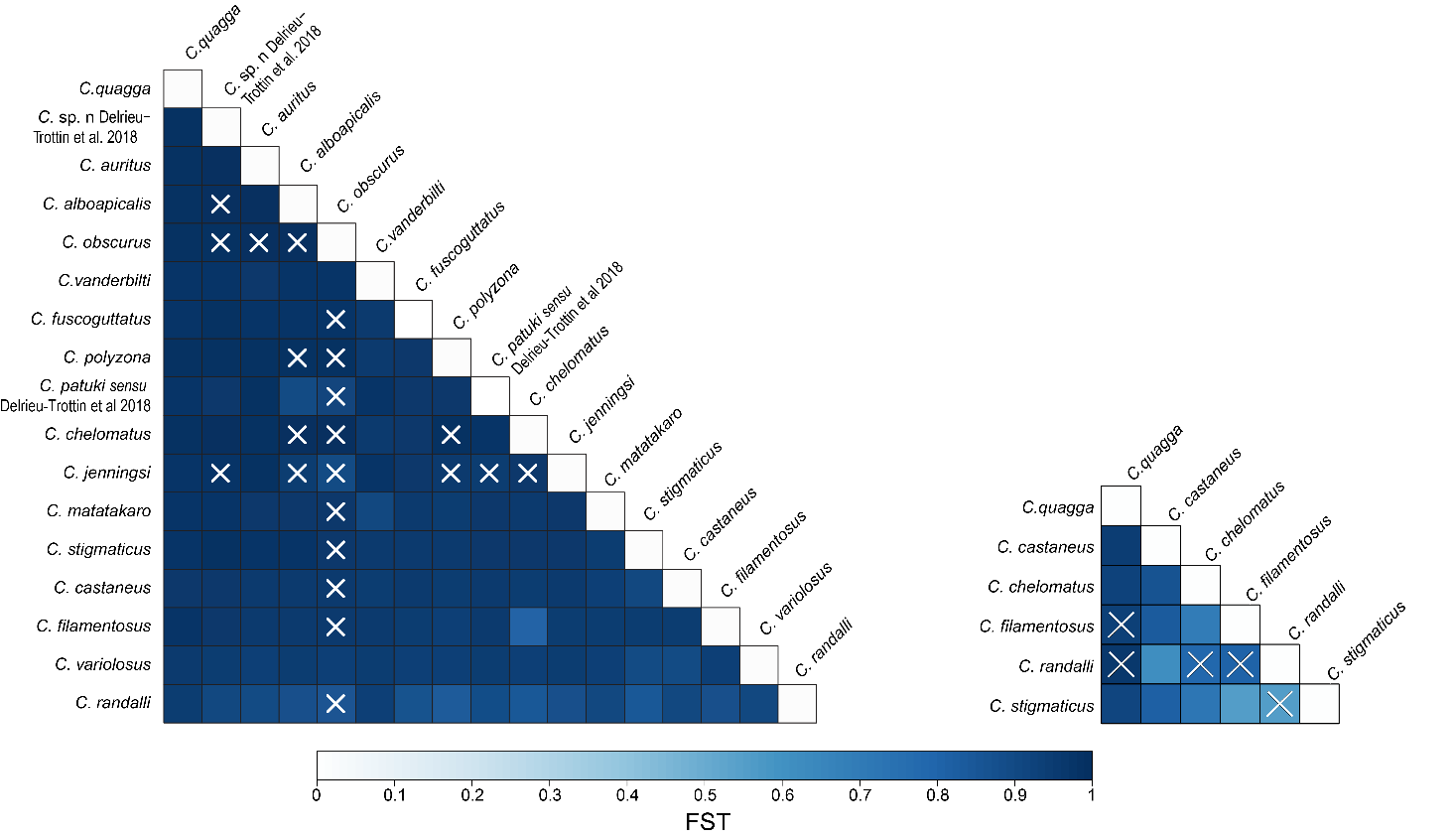


Appendix 9: Pairwise FST values for the mtDNA COI of the 17 *Cirripectes* species and associated p-values (below diagonal).

|  | *C. alboapicalis* | *C. auritus* | *C. castaneus* | *C. chelomatus* | *C. filamentosus* | *C. fuscoguttatus* | *C. jenningsi* | *C. matatakaro* | *C. obscurus* | *C. patuki sensu* Delrieu-Trottin et al. 2018 | *C. polyzona* | *C. quagga* | *C. randalli* | *C.*sp.n. Delrieu-Trottin | *C. stigmaticus* | *C. vanderbilti* | *C. variolosus* |
| --- | --- | --- | --- | --- | --- | --- | --- | --- | --- | --- | --- | --- | --- | --- | --- | --- | --- |
| *C. alboapicalis* |  | 0.99329 | 0.95542 | 0.9941 | 0.95872 | 0.97644 | 0.94488 | 0.96944 | 1 | 0.89362 | 0.98713 | 0.98188 | 0.87097 | 0.99331 | 0.97654 | 0.97387 | 0.94125 |
| *C. auritus* | 0.01802 |  | 0.95028 | 0.98696 | 0.95849 | 0.97814 | 0.98213 | 0.96108 | 0.99287 | 0.98134 | 0.98667 | 0.98625 | 0.89276 | 0.99428 | 0.97662 | 0.96309 | 0.93859 |
| *C. castaneus* | 0.00000 | 0.00000 |  | 0.94098 | 0.94122 | 0.95088 | 0.95692 | 0.93973 | 0.95334 | 0.95759 | 0.94039 | 0.96196 | 0.90256 | 0.96106 | 0.90066 | 0.94851 | 0.89152 |
| *C. chelomatus* | 0.11712 | 0.01802 | 0.00000 |  | 0.80526 | 0.96583 | 0.9657 | 0.95286 | 0.99103 | 0.97171 | 0.98039 | 0.98256 | 0.8488 | 0.99407 | 0.96653 | 0.95721 | 0.93091 |
| *C. filamentosus* | 0.02703 | 0.00000 | 0.00000 | 0.00901 |  | 0.94345 | 0.94844 | 0.94423 | 0.95246 | 0.95679 | 0.93969 | 0.97305 | 0.87216 | 0.96783 | 0.9459 | 0.95033 | 0.93176 |
| *C. fuscoguttatus* | 0.02703 | 0.00000 | 0.00000 | 0.01802 | 0.00000 |  | 0.96357 | 0.95343 | 0.97051 | 0.96742 | 0.96367 | 0.97988 | 0.86296 | 0.98312 | 0.95885 | 0.95639 | 0.93571 |
| *C. jenningsi* | 0.29730 | 0.02703 | 0.00901 | 0.05405 | 0.01802 | 0.01802 |  | 0.96912 | 0.89781 | 0.94664 | 0.95705 | 0.97722 | 0.87057 | 0.97504 | 0.96328 | 0.97322 | 0.94287 |
| *C. matatakaro* | 0.00000 | 0.00000 | 0.00000 | 0.00000 | 0.00000 | 0.00000 | 0.00901 |  | 0.96794 | 0.96783 | 0.94896 | 0.97277 | 0.90559 | 0.97358 | 0.94592 | 0.90351 | 0.93768 |
| *C. obscurus* | 0.99099 | 0.99099 | 0.99099 | 0.99099 | 0.99099 | 0.99099 | 0.99099 | 0.99099 |  | 0.91979 | 0.98039 | 0.98081 | 0.85403 | 0.9898 | 0.96759 | 0.97415 | 0.93532 |
| *C. patuki sensu* Delrieu-Trottin et al. 2018 | 0.04505 | 0.00000 | 0.00000 | 0.03604 | 0.00000 | 0.00000 | 0.05405 | 0.00000 | 0.10811 |  | 0.96793 | 0.97631 | 0.88916 | 0.96586 | 0.96942 | 0.97281 | 0.947 |
| *C. polyzona* | 0.12613 | 0.00000 | 0.00000 | 0.15315 | 0.00000 | 0.01802 | 0.15315 | 0.00000 | 0.30631 | 0.02703 |  | 0.98363 | 0.83894 | 0.9899 | 0.95795 | 0.95505 | 0.93 |
| *C. quagga* | 0.00901 | 0.00000 | 0.00000 | 0.00000 | 0.00000 | 0.00000 | 0.00000 | 0.00000 | 0.03604 | 0.00000 | 0.00000 |  | 0.94077 | 0.98345 | 0.97979 | 0.97444 | 0.95102 |
| *C. randalli* | 0.00901 | 0.00000 | 0.00000 | 0.00000 | 0.00000 | 0.00901 | 0.00000 | 0.00000 | 0.08108 | 0.00000 | 0.00901 | 0.00000 |  | 0.90649 | 0.84802 | 0.93917 | 0.90611 |
| *C.*sp. n. Delrieu-Trottin et al. 2018 | 0.06306 | 0.00901 | 0.00000 | 0.02703 | 0.00000 | 0.00901 | 0.06306 | 0.00000 | 0.09910 | 0.01802 | 0.00901 | 0.00000 | 0.00000 |  | 0.98112 | 0.97348 | 0.94878 |
| *C. stigmaticus* | 0.02703 | 0.00000 | 0.00000 | 0.00000 | 0.00000 | 0.00000 | 0.02703 | 0.00000 | 0.09009 | 0.00000 | 0.01802 | 0.00000 | 0.00000 | 0.00000 |  | 0.95202 | 0.8914 |
| *C. vanderbilti* | 0.00000 | 0.00000 | 0.00000 | 0.00000 | 0.00000 | 0.00000 | 0.00000 | 0.00000 | 0.00000 | 0.00000 | 0.00000 | 0.00000 | 0.00000 | 0.00000 | 0.00000 |  | 0.9493 |
| *C. variolosus* | 0.00000 | 0.00000 | 0.00000 | 0.00000 | 0.00000 | 0.00000 | 0.00000 | 0.00000 | 0.01802 | 0.00000 | 0.00000 | 0.00000 | 0.00000 | 0.00000 | 0.00000 | 0.00000 |  |

Appendix 10: Median/joining networks showing the relationships among nuclear rhodopsin haplotypes in *Cirripectes* species. Each circle represents a haplotype with its size proportional to its total frequency. Black crossbars on branches indicate single nucleotide changes, black nodes represent unsampled probable haplotypes, colours indicate collection location.


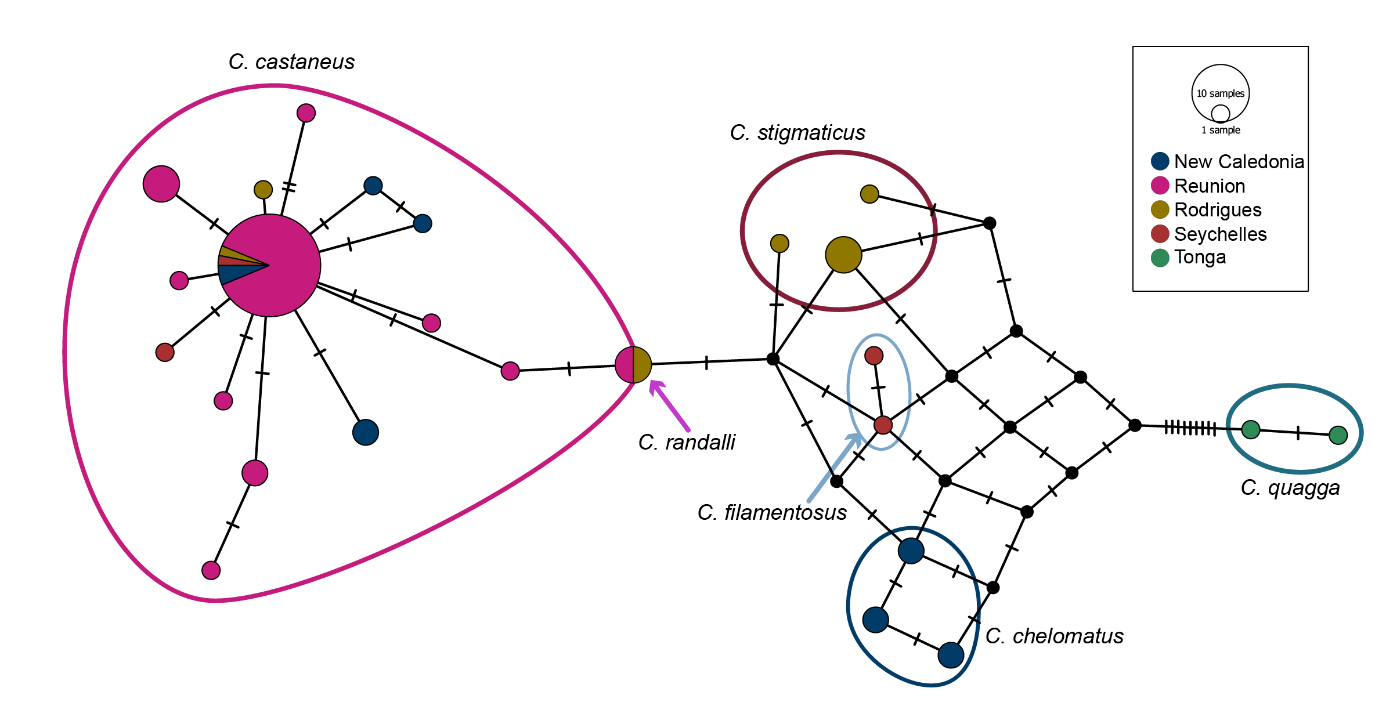


Appendix 11: Detailed description of *Cirripectes* mitogenome.

The mitogenome lengths are within the ranges of other teleost mitogenomes. As in other vertebrates (Miya et al. 2001), they contained 13 protein-coding genes, 2 rRNA genes (12S and 16S), 22 tRNA, and a control region (Appendix  11.1). Among the tRNAs, two forms of tRNA-Leu and tRNA-Ser were identified as in other bony fishes (Prosdocimi et al., 2012). Likewise, most mitochondrial genes were encoded on the H-strand, with only ND6 and eight tRNA (Gln, Ala, Asn, Cys, Tyr, Ser [only one of the two tRNA-Ser], Glu, and Pro) genes encoded on the L-strand (Wang et al., 2021). The ATPase 6 and ATPase 8 overlapped by 10 nucleotides, and ND4 and ND4L shared 7 nucleotides. ND5 and ND6 overlapped by 9 nucleotides (but only 4 nucleotides for RUNA_0737) on the opposite strand. The 12S ribosomal genes are 948 bp long for the three species (Appendix 11.2). The 16S ribosomal RNA genes showed some individual length variation among *C. castaneus* with 1,684 bp in 18 individuals, but 1,685 bp in one (RUNA_4002; T insertion at 1,343 bp). For *C. randalli* and *C. stigmaticus*, the 16S ribosomal sequences are 1,681 bp and 1,682 bp long, respectively. They were located between tRNA-Phe and tRNA-Leu, and were separated by tRNA-Val, as they were in other vertebrates (Miya et al., 2003) The 22 tRNA genes were interspersed in the genome and ranged in size from 65 to 75 bp.

Appendix 11.1: Visualization and annotation using Mitofish (Sato et al., 2018) of the mitochondrial genome of *Cirripectes stigmaticus*. Black = protein coding sequences, Red = tRNAs, Tan = rRNAs, Brown = control region.


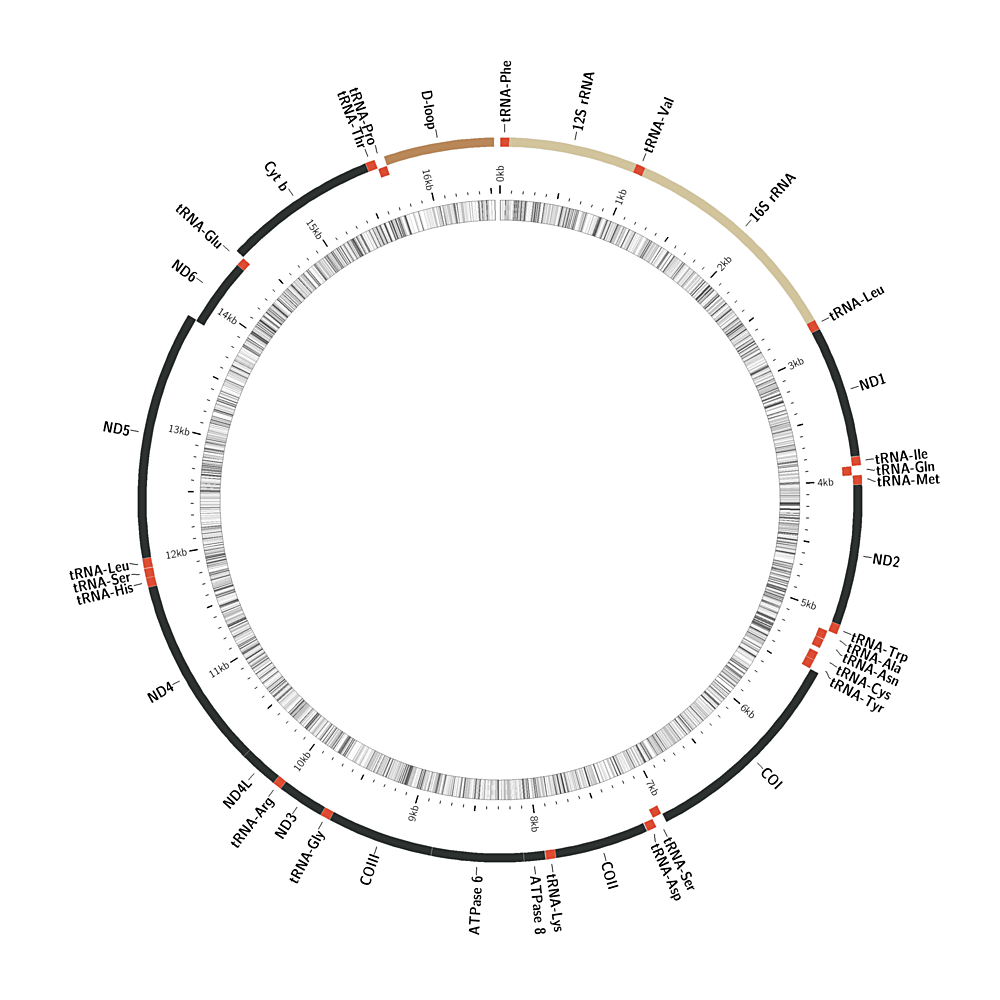


Appendix 11.2: Length of mitochondrial genes found in *C. castaneus*, *C. stigmaticus* and *C. randalli*. In grey, genes with intra- and inter-species length variability.

| **Gene in position order** | **Type** | **Strand** | **Length in bp for each species** | | |
| --- | --- | --- | --- | --- | --- |
|  |  |  | ***C. castaneus* (N=19)** | ***C. stigmaticus* (N=4)** | ***C. randalli* (N=1)** |
| tRNA/Phe | transfer RNA | H |  | 69 |  |
| 12S | ribosomal RNA | H |  | 948 |  |
| tRNA/Val | transfer RNA | H |  | 72 |  |
| 16S | ribosomal RNA | H | 1,684 (18) ; 1,685 (1) | 1,682 | 1,681 |
| tRNA/Leu | transfer RNA | H |  | 75 |  |
| ND1 | protein coding | H |  | 975 |  |
| tRNA/Ile | transfer RNA | H |  | 69 |  |
| tRNA/Gln | transfer RNA | L |  | 71 |  |
| tRNA/Met | transfer RNA | H |  | 70 |  |
| ND2 | protein coding | H |  | 1,042 |  |
| tRNA/Trp | transfer RNA | H |  | 73 |  |
| tRNA/Ala | transfer RNA | L |  | 69 |  |
| tRNA/Asn | transfer RNA | L |  | 73 |  |
| tRNA/Cys | transfer RNA | L |  | 65 |  |
| tRNA/Tyr | transfer RNA | L |  | 70 |  |
| COI | protein coding | H |  | 1,572 |  |
| tRNA/Ser | transfer RNA | L |  | 71 |  |
| tRNA/Asp | transfer RNA | H |  | 73 |  |
| COII | protein coding | H |  | 691 |  |
| tRNA/Lys | transfer RNA | H |  | 74 |  |
| ATPase8 | protein coding | H |  | 168 |  |
| ATPase6 | protein coding | H |  | 683 |  |
| COIII | protein coding | H |  | 785 |  |
| tRNA/Gly | transfer RNA | H | 72 | 73 | 73 |
| ND3 | protein coding | H |  | 349 |  |
| tRNA/Arg | transfer RNA | H |  | 68 |  |
| ND4L | protein coding | H |  | 297 |  |
| ND4 | protein coding | H |  | 1,381 |  |
| tRNA/His | transfer RNA | H |  | 69 |  |
| tRNA/Ser | transfer RNA | H |  | 68 |  |
| tRNA/Leu | transfer RNA | H |  | 73 |  |
| ND5 | protein coding | H | 1,827 (1); 1,842 (14);1,863(4) | 1,842 | 1,842 |
| ND6 | protein coding | L |  | 522 |  |
| tRNA/Glu | transfer RNA | L |  | 68 |  |
| Cyt b | protein coding | H |  | 1,141 |  |
| tRNA/Thr | transfer RNA | H |  | 72 |  |
| tRNA/Pro | transfer RNA | L |  | 70 |  |
| Dloop |  | H | 820/825 | 818/820 | 818 |
| Total Length |  |  | 16,476 / 16,512 | 16,482 / 16,532 | 16,482 |

Appendix 12: Pictures of *Cirripectes* species sampled in the Mascarene Archipelagos using ARMS: *C. castaneus*: a: RUNA_3256, b: RUNA_0854 and c: RUNA_2719; *C. randalli*: d: ORCIE1990 and e: RUNA_3176; and *C. stigmaticus*: f: 1583 and g: ORCIE1889. Pictures were edited in Adobe Photoshop CS6.


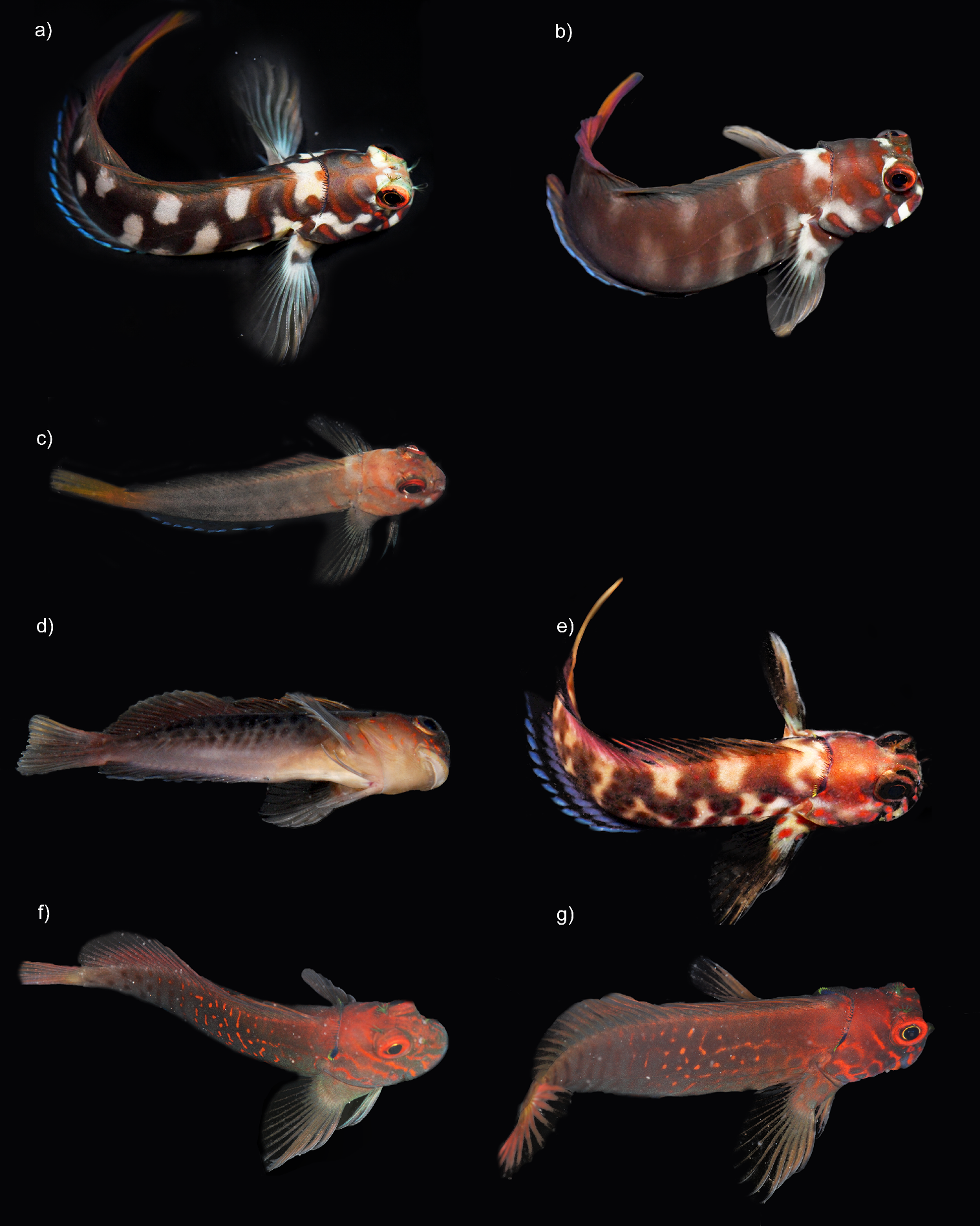

Supplement: Supplementary file 1 — Appendix S1–S12 [file ECE3-13-e9850-s001.docx]
